# Tree Seedling‐Recruitment in Temperate and Subtropical Species: A Systematic Narrative Review of Biotic and Abiotic Modifiers, and Experimental Treatments

**DOI:** 10.1002/ece3.73399

**Published:** 2026-04-10

**Authors:** Sarah Bürli, Hannah L. Buckley, Bradley S. Case

**Affiliations:** ^1^ School of Science | Te Kura Putaiao Auckland University of Technology Auckland New Zealand; ^2^ Botanical Garden, Institute for Biology University of Graz Graz Austria

**Keywords:** biotic and abiotic modifiers, germination, seed priming techniques, seedling recruitment, seedling survival and growth, temperate and subtropical tree species

## Abstract

Tree seedling recruitment, encompassing germination and growth, is crucial for natural and artificial propagation. Recruitment depends on the interplay of biotic and abiotic modifiers and can be influenced by experimental treatments and nursery methodologies, such as seed priming techniques and seedling treatments, which affect the success of forest restoration and nursery operations. Despite extensive research, the effects of natural modifiers and treatments across species remain poorly understood. We conducted a systematic review of 91 peer‐reviewed articles to identify patterns and knowledge gaps on the effects of natural modifiers, seed priming, and seedling treatments on seedling recruitment. Our Scopus search focused on experimental studies of tree germination and seedling fitness in temperate and subtropical species. Starting with 266 articles, we critically appraised them for internal validity using two sets of criteria and independent evaluators. Data on tree responses of studies with internal validity were categorized and analyzed narratively. Most reviewed studies (60.5%) focused on abiotic modifiers. Seedling‐focused studies (66.6%) outnumbered seed‐focused ones (33.4%). Overall, 42.2% of the studies reported nonsignificant effects. Fertilization, temperature, precipitation, pollution, and light availability had mostly nonsignificant effects on seedling recruitment. Soil moisture, snow cover, and soil scarification had positive effects, while shading negatively impacted seedlings. Mixed biotic and abiotic modifiers showed inconclusive results. Mycorrhizal associations promoted recruitment, while competition had mixed effects. Seed stratification was more effective than scarification or chemical treatments in promoting germination. To advance understanding of tree recruitment processes, we recommend studies on seedling treatments, mycorrhizal associations, fertilization, warm stratification, scarification, and chemical treatment across species. Frequent nonsignificant results suggest reassessing ecological hypotheses, incorporating context‐specific variables, and adopting more robust experimental designs. A trait‐based approach seems essential for identifying broader patterns and enhancing conservation and restoration outcomes.

## Graphical abstract

Our systematic review of 91 peer‐reviewed articles on tree recruitment highlights a research bias toward seedling‐focused studies and reveals a high prevalence of nonsignificant results. We recommend targeted experimental work on seedling treatments, mycorrhizal associations, fertilization, warm stratification, scarification, and chemical treatments across a wide range of species. A trait‐based approach is essential to uncover generalizable patterns and improve the effectiveness of ecological restoration and conservation efforts.

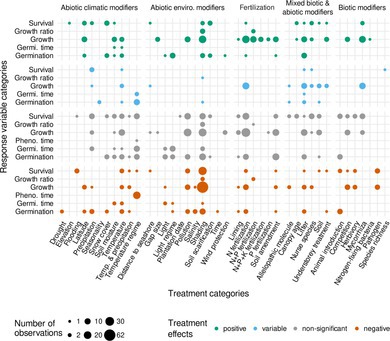

## 1 Introduction

Trees play an important role in global biodiversity, providing ecological, cultural, and economic benefits (Nilsson et al. 2011). Seedling recruitment, which includes seed germination and early growth and establishment, is the primary means for both natural and artificial tree propagation (Berjak and Pammenter 2004) and is crucial for genetic transmission, plant fitness, species persistence, and forest composition (Grime and Hillier 1992). However, it can be a major bottleneck, impacting forest dynamics and distribution (Ibáñez et al. 2007; Vanderwel et al. 2013). Successful recruitment is especially important in restoration efforts, particularly for species with low survival rates in the wild. Consequently, research has focused on understanding the factors affecting seedling recruitment in both natural and controlled environments.

Seedling‐establishment success depends on the interplay of various ecological processes driven by biotic and abiotic natural modifiers, which can either enhance or filter seedling recruitment (Das and Biswas 2022). Biotic modifiers include interactions among organisms, such as competition, herbivory, seed predation, microbial pathogenicity, and mutualistic symbiosis (e.g., Martin et al. 2017; Yin et al. 2023), while abiotic modifiers encompass factors like temperature, soil moisture, light availability, and soil physico‐chemistry (e.g., BassiriRad et al. 2015; Galíndez et al. 2019). Natural modifiers that exert both abiotic and biotic effects, hereafter referred to as mixed biotic‐abiotic modifiers, such as nurse species, influence seedling recruitment by both competing with seedlings and enhancing their microclimatic conditions (e.g., Dupuy and Chazdon 2008).

In addition to natural modifiers, various experimental and artificial treatments have been developed to enhance seedling success in propagation and planting contexts (e.g., McConnaughay et al. 1996; Connolly et al. 2017; Galíndez et al. 2019). These treatments fall into two main categories: seed priming techniques and seedling treatments. Seed priming is pre‐sowing treatments that activate physiological and biochemical processes in seeds, including enhanced enzyme activity, antioxidant accumulation, hormone modulation, and cellular repair (Rhaman 2025), thereby improving germination rates and seedling vigor (Nawaz et al. 2013; Waqas et al. 2019). Seed priming encompasses traditional techniques such as stratification and scarification (e.g., Connolly et al. 2017; Lacoretz et al. 2022), but also more recent technological advances, including nano‐priming (Rhaman 2025; Stefanello et al. 2026). In contrast, seedling treatments, such as seedling transplantation (e.g., Velazco et al. 2018), are applied to young plants after germination. Many of these techniques are commonly used in tree nurseries, botanical gardens, and conservation initiatives (Sisodia et al. 2018; Iralu et al. 2019; personal observations).

Understanding how natural modifiers affect tree recruitment and early fitness is key to predicting forest dynamics under global change (Canham and Murphy 2016). Additionally, knowing how experimental treatments influence seedling recruitment is essential for improving forest restoration and nurseries' efficiency. Both abiotic and biotic modifiers impact recruitment in a species‐dependent way, such as fertilization and the inoculation of mycorrhizae or their removal by soil sterilization (e.g., Willis and Walters 2018; Zhang et al. 2019; Liang et al. 2022; see also Crofts and Brown 2020; Ibáñez et al. 2022). Similarly certain experimental methods, such as cold and warm stratification, only affect specific species (e.g., Morgenson 2000; Baskin et al. 2002; Connolly et al. 2017). Despite many studies, the consistency of these effects across species is still unclear (but see Iralu et al. 2019 for a review on seed germination in threatened species). Therefore, it is important to summarize what is known about the impacts of natural modifiers and experimental treatments on tree recruitment.

To synthesize the effects of biotic and abiotic modifiers, seed priming techniques, and seedling treatments across several tree species, we conducted a systematic narrative review of 91 studies. Our goals were to identify patterns in how these modifiers influence recruitment in tree species from temperate and subtropical biomes, and identify research gaps to set an agenda of required future investigations into recruitment processes. We asked and answered the following three research questions: (1) What effects do biotic and abiotic modifiers have on recruitment of tree seedlings? (2) What seed priming and seedling treatment techniques are most prevalent and what are their effects on seedling recruitment parameters? (3) What knowledge gaps remain, and what directions should future research take?

## 2 Method

We searched the international literature for relevant, peer‐reviewed articles detailing primary experimental research on the effects of seed priming techniques, seedling treatments, and biotic and abiotic modifiers on seedling recruitment in tree species. Our focus was on species from temperate and subtropical biomes, which encompass a significant portion of the Earth's land surface between the Tropics (23°27′) and the polar circles (66°33′; Cui et al. 2021). To structure our review scope, we developed and used the following PICO‐model (Slodowicz et al. 2019): Population: Seeds and seedlings of tree species (excluding shrub and tall grass species, e.g., bamboos) from temperate and subtropical biomes; Intervention: Seed priming and/or seedling treatments, and biotic and/or abiotic modifiers modifying seed germination and seedling fitness and establishment; Comparator: Absence of treatment; natural or different levels of a modifier in the environment; Outcome: Changes in dormancy break, seed viability, germination (e.g., percentage) and/or seedling fitness traits (e.g., survival, performance, mass).

Using our PICO model as a framework and following the recommendations of the Collaboration for Environmental Evidence (CEE) Guidelines (Pullin et al. 2018), we crafted a search string (Material S1) to identify relevant papers aligned with our review scope. The search query was refined through iterative adjustments in the Scopus database (https://www.scopus.com/) and by incorporating keywords found in papers selected from earlier versions of the search string. Feedback from colleagues further enhanced the precision of the search. On August 15, 2023, our search in the Scopus database yielded a total of 266 papers (Material S2). After removing two duplicates, we reviewed the abstracts and applied the following inclusion criteria: studies (a) on tree species from temperate and subtropical biomes, (b) that tested the effects of at least one modifier or treatment, and (c) that included seed germination or dormancy, or seedling, germinant or juvenile establishment, performance, growth, fitness, or survival as response variables. As a result, 135 papers were excluded, leaving 131 for full‐text screening. Each paper was then thoroughly evaluated against the initial inclusion criteria, as well as four additional criteria: (a) availability of the full text in English (eight papers were excluded due to being published only in Chinese), (b) being empirical and experimental (excluding purely observational and modeling studies, reviews, and meta‐analyses), (c) having a sufficient sample size to support inferences (only one paper, Sánchez‐Gómez et al. 2006, was excluded based on this criterion), and (d) including experimental controls with clearly reported levels, strength, duration, and concentration of treatments. In our review, a “paper” refers to a published article, while a “study” denotes a research experiment within a paper, allowing for one paper to encompass multiple studies. To ensure consistency in the selection process, a second evaluator independently appraised a subset of 25% of the retained papers (33 out of 131) for inclusion. Ultimately, 40 papers were excluded during full‐text screening, resulting in 91 papers included in our final analysis. All included papers were published between November 1961 and June 2023. From each included paper, we extracted a range of variables and information, including research aims, study types (experimental, observational, review, or meta‐analysis), reasons for exclusion, treatments and modifiers investigated along with their control treatments, levels (e.g., concentration) and effects, response variables examined, corresponding life stages (i.e., seed or seedling), reported co‐variables influencing treatment effects, interaction effects of treatments and modifiers, taxa studied, and study locations. This process resulted in 104 independent studies across the 91 research papers, from which we extracted 1126 observations. However, 12 observations were excluded from further analysis due to unclear reporting of the effects of treatments or modifiers on the response variables, leading to a final dataset of 1114 observations for analysis.

We defined “response variables” of interest prior to screening, based on their relevance to the review scope and their prevalence in the literature. We extracted data solely for the selected “response variables” (Table 1). This approach allows an efficient comparison across studies, but means our review does not encompass all response variables reported in the screened papers. Excluded response variables were biomass ratios of two plant compartments (but biomass ratios of a plant compartment compared to the whole plant were included), such as leaf:stem, stem:root, leaf:root, belowground:aboveground ratios (e.g., Lu et al. 2018), and variables linked to plant respiration, leaf water potential, water use efficiency (WUE; e.g., Lu et al. 2022), leaf size, SLA and leaf chlorophyll content (SPAD; e.g., Niu et al. 2011).

**TABLE 1:** Response variable categories and the a priori defined response variables.

Response variable category	Life stages	Response variables
Biomass ratio	Seedling	Leaf biomass % and ratio, stem biomass % and ratio, root biomass % and ratio—each related to total plant biomass
Germination	Seed	Germination %, density, probability, proportion, and rate, viable seed %
Germination time	Seed	Germination time
Growth	Seedling	Above biomass, above biomass increment, leaf biomass, shoot biomass, stem biomass, root biomass, total plant biomass, height, height increment, stem diameter increment, diameter at breast level, basal diameter, root collar diameter, root diameter, radicle length, root length, growth rate, height growth rate, relative growth rate, relative height growth rate, relative biomass growth rate
Phenology	Seedling	Time to budburst, time to leafout
Survival	Seedling	Binary survival, survival %, density, probability, proportion, rate, and time

**FIGURE 1:**
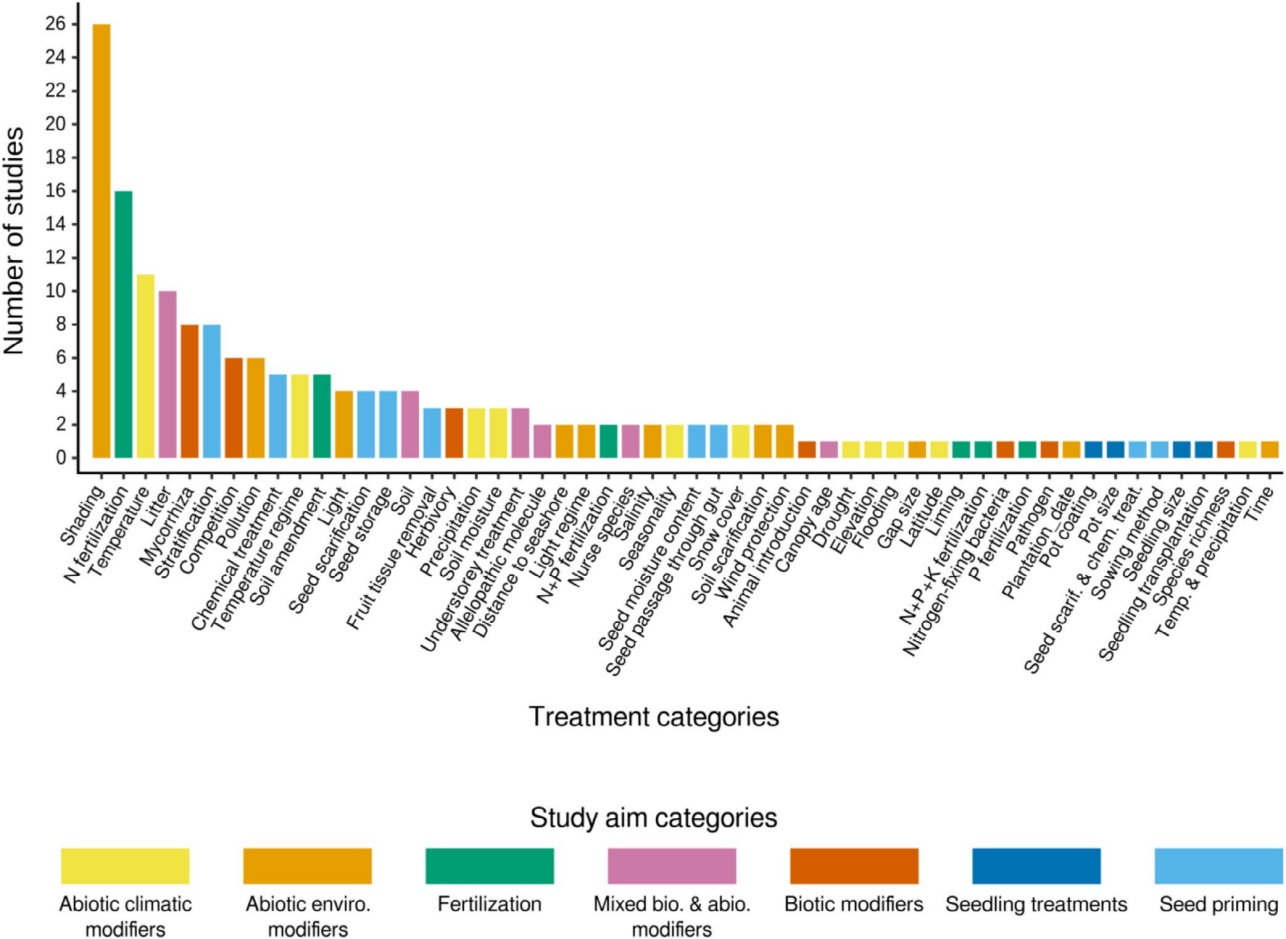
Total number of reviewed studies, categorized by treatment category with colors representing different study aim categories.

For each study, we categorized the tested modifiers and treatments into specific “treatment categories.” Subsequently, these treatment categories were grouped into broader “study aim categories,” based on whether they focused on abiotic or biotic modifiers, seed or seedling treatments (Table 2). In total, we examined the effects of 54 treatment categories, organized into seven defined study aim categories, across the 104 studies included in our review (Figure 1; Table 2). Response variables were similarly organized into six broad categories, referred to as “response variable categories,” based on life stages (e.g., seed and seedling) and the type of response measured. For example, germination percentage and density were grouped together because improvements in both metrics indicate a higher number of germinants (Table 1). This categorization is a valuable method for inferring general patterns regarding the effects of various treatments or modifiers on a set of response variables.

**TABLE 2:** Study aim categories with their definition and the treatment categories included within each of them.

Study aim categories	Definition	Treatment categories
Abiotic climatic modifiers	Abiotic modifier associated with climatic variables	Drought, elevation, flooding (e.g., duration), latitude, precipitation, seasonality, snow cover, soil moisture, temperature (increase), temperature + precipitation, temperature regime (*n =* 11; *n* studies = 27)
Abiotic environmental modifiers	Abiotic modifiers not associated with climatic variables	Distance to seashore, gap size in forest, light, light regime, plantation date, pollution, salinity, shading, soil scarification, time (e.g., since plantation), wind protection (*n =* 11; *n* studies = 43)
Biotic modifiers	Biotic modifiers	Animal introduction, competition (i.e., intra‐ and/or interspecific competition and plant density), herbivory (e.g., predation and defoliation, human‐made simulated herbivory), mycorrhiza (inoculation or removal), nitrogen‐fixing bacteria inoculation, pathogens inoculation, plant species richness (*n =* 7; *n* studies = 19)
Fertilization	Amendment of soil for the purpose of enhancing seed/seedling conditions	Liming, N fertilization N‐P fertilization, N‐P‐K fertilization, P fertilization, total soil amendment (ash, biochar, or charcoal amendment) (*n =* 6; *n* studies = 25)
Mixed biotic & abiotic modifiers	Modifier implying both biotic and abiotic processes	Allelopathic molecule, canopy age, plant litter addition, nurse species, soil (origin and/or type and soil sterilization), understorey treatment (e.g., removal or control of understorey vegetation and/or weeds) (*n =* 7; *n* studies = 19)
Seed priming techniques	Treatments of seeds before germination	Fruit tissue removal, seed chemical treatment, seed moisture content, seed passage through animal gut, seed scarification, seed scarification + chemical treatment, seed sowing method (e.g., germination container type), seed storage (time and/or condition), seed stratification (*n = 9*; *n* studies = 20)
Seedling treatments	Seedling treatments not covered by the other categories	Pot coating, pot size, seedling size, seedling transplantation method (*n =* 4; *n* studies = 4)

*Note:* The number of treatment categories (*n*) and reviewed studies (*n* studies) per study aim categories are given in brackets in the treatment categories column.

After evaluating the feasibility of a quantitative meta‐analysis in accordance with the Cochrane Handbook for Systematic Reviews of Interventions (Chandler et al. 2019), we determined that statistical pooling was not appropriate due to substantial methodological heterogeneity, weakly comparable outcomes, and insufficient numerical data for effect size calculation. Accordingly, we conducted a systematic narrative review (Littell et al. 2008), enhancing analytical rigor by structuring the evidence into comparative tables and applying vote counting based on direction of effect (Chandler et al. 2019). This approach provides a transparent, systematic synthesis while remaining aligned with the nature of the available evidence.

To conduct the vote counting based on effect direction (Chandler et al. 2019), we standardized the treatment effects on response variables to be either “positive,” “variable,” “non‐significant,” or “negative.” The designation “variable” was used when the paper authors or our analysis found no clear trend. “Positive” and “negative” effects were defined based on the treatments' impact on the response variable, indicating an improvement or decline, respectively, compared to the control treatment. The designation “non‐significant” was applied when statistical analyses revealed no significant effect of the treatment on the response variable. Analyses and graphs were performed using R 4.1.2. (R Core Team 2021) with the packages “ggplot2” and “dplyr” (Wickham 2016; Wickham et al. 2023).

## 3 Summary of Reviewed Studies, Response Variables, and Treatment Effects

**FIGURE 2:**
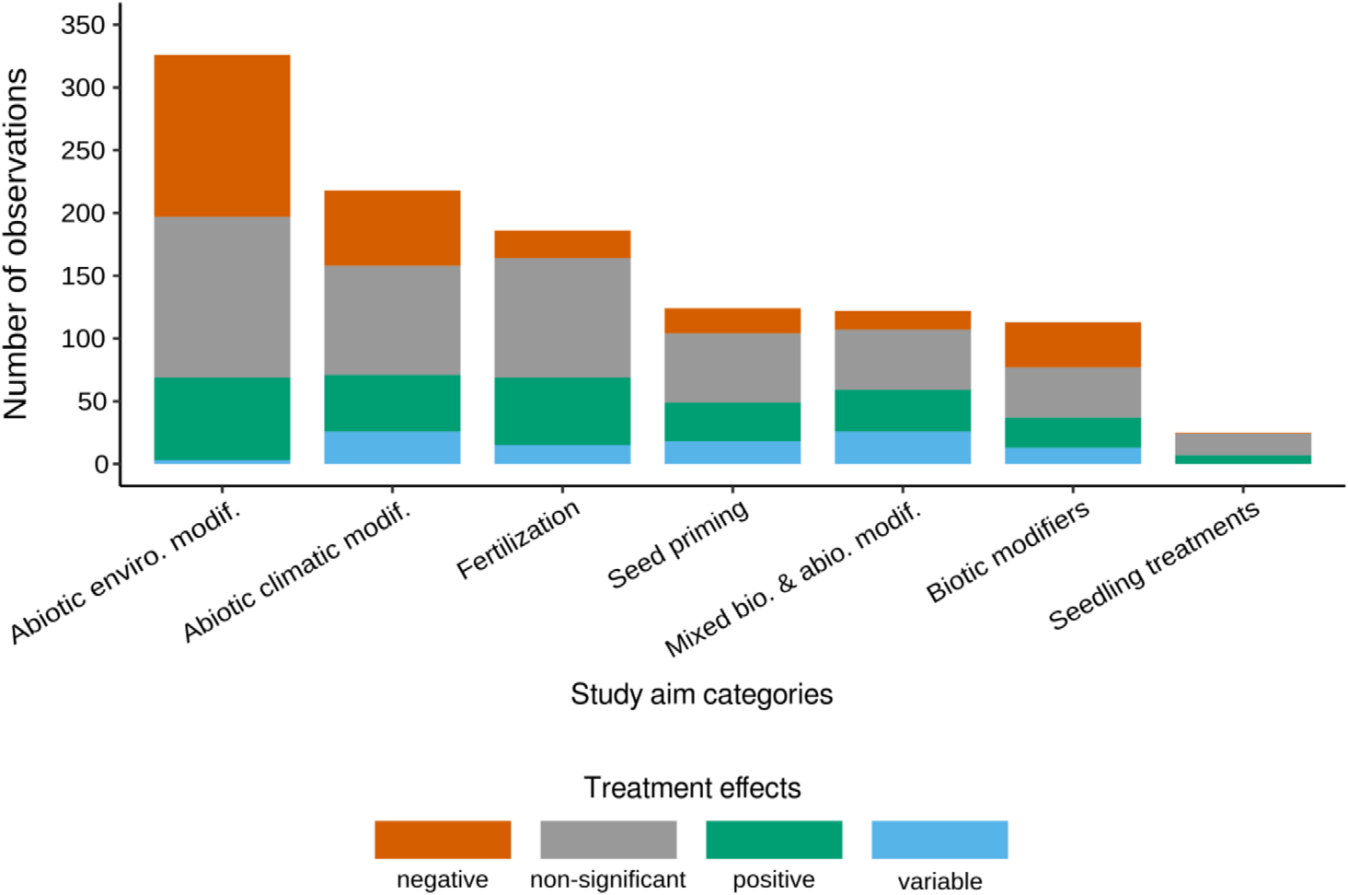
Observation number by study aim category, with treatment effects represented by distinct colors: Negative effects in orange, nonsignificant effects in gray, positive effects in green, and variable effects in blue.

Six of the 54 treatment categories—shading, N‐fertilization, temperature, litter addition, mycorrhizae, and seed stratification—accounted for 37.7% of the total studies examined. Overall, the majority of studies (60.5%) concentrated on abiotic modifiers, encompassing climatic, environmental, and fertilization modifiers (Table 2). The largest portion of the observations (42.2%; *n* = 470) from the reviewed studies yielded nonsignificant effects. In comparison, 283 observations indicated negative effects, 260 indicated positive effects, and 101 indicated variable effects. This prevalence of nonsignificant effects was consistent across all study aim categories (Figure 2), except for abiotic environmental modifiers, where negative effects outnumbered nonsignificant ones by one.

**FIGURE 3:**
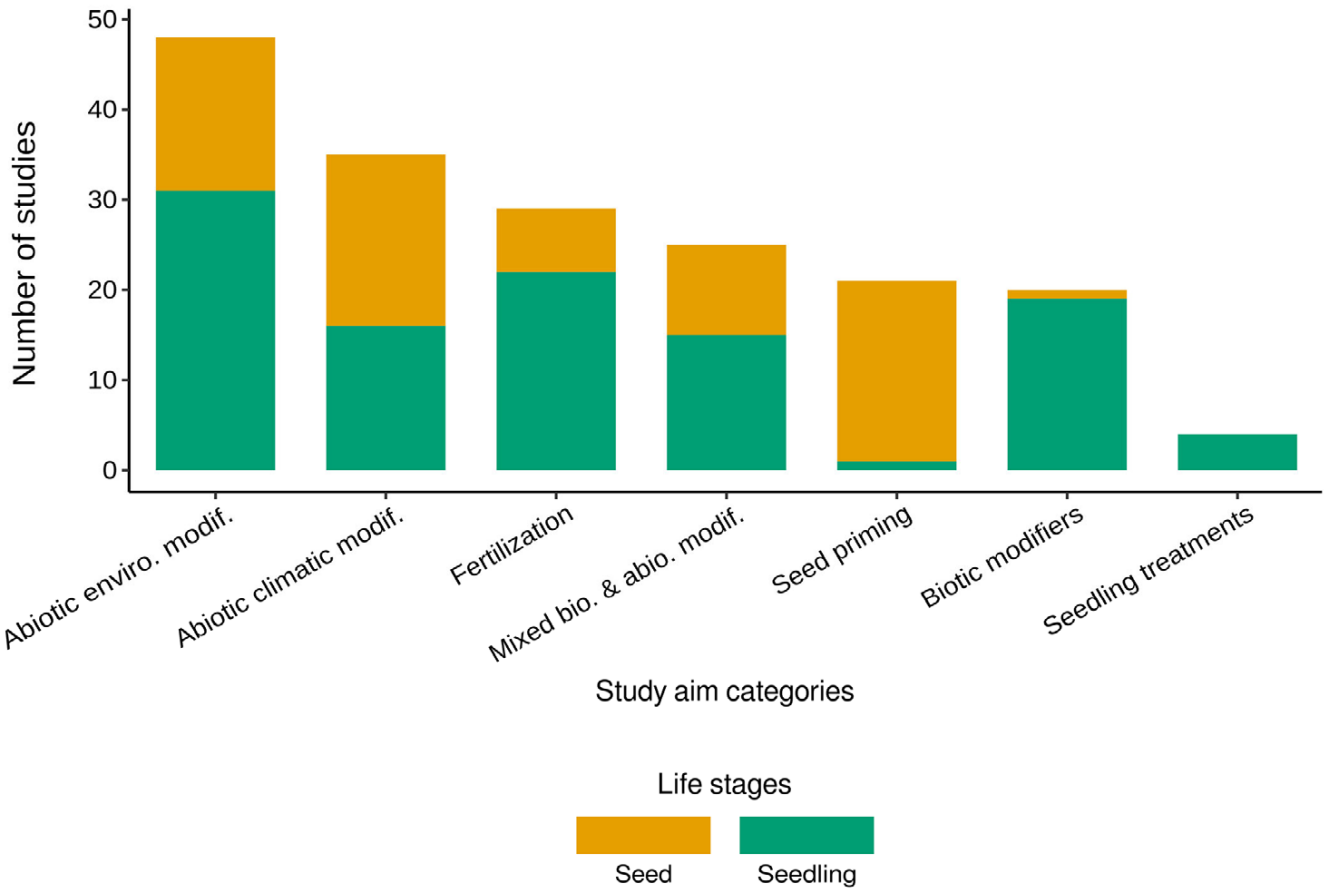
Number of reviewed studies per study aim category on seeds (orange) and seedlings (green).

Reviewed studies were predominantly seedling‐focused (66.6%, *n =* 742 observations of 1114), with fewer studies focusing on seeds (33.4%, *n =* 372 observations of 1114; Figure 3). Unlike other categories of natural modifiers, where over 70% of observations focused on seedlings, studies on abiotic climatic modifiers displayed a more balanced distribution, with 40.5% of observations on seeds and 59.5% on seedlings. All studies investigating seedling treatment effects were exclusively conducted on seedlings, while studies on seed priming techniques targeted seeds with one notable exception investigating the effects of seed chemical treatment on seedling growth (Li et al. 2017). This trend is reflected in the response variables evaluated in our review, with seedling growth and survival being two of the three most frequently examined response variables, in 37.9% and 25.3% of the studies, respectively (germination, germination time, seedling biomass ratio and phenology were investigated in 28.2%, 6.32%, 1.71%, and 0.57% of the studies, respectively).

The predominant focus on seedlings largely stems from the understanding that numerous treatments, such as competition, presence of nurse species, gap sizes in forest canopies and fertilization, tend to have a greater impact on seedling establishment and growth compared to seed germination. Moreover, seedlings typically exhibit more visible and immediate responses to environmental factors, making their effects easier to measure and analyze. However, this emphasis on seedlings leaves important questions about the processes affecting seeds themselves. Gaining insights into how these modifiers influence seed viability and germination could enhance our understanding of plant population dynamics and community structure.

## 4 What Effects Did Biotic and Abiotic Modifiers Have on Recruitment of Tree Seedlings?

### 4.1 Fertilization—No Consistent Recruitment Benefit From Fertilization Treatments

**FIGURE 4:**
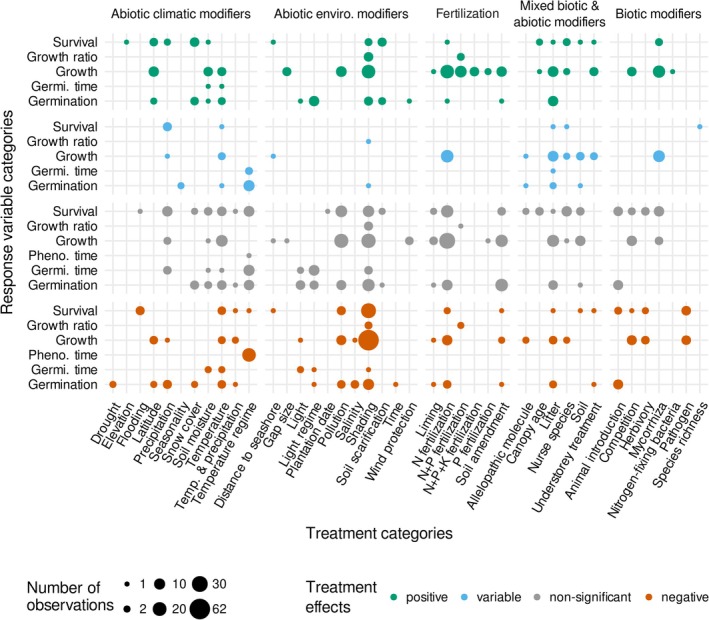
Cross‐tabulation results for the 80 reviewed studies (across 69 papers) that investigated effects of 42 biotic and abiotic treatment categories (*x*‐axis) grouped in the five study aim categories (displayed as vertical panels; except seed priming technique and seeding treatment categories) tested on seed germination and germination time, seedling growth, growth ratio, and survival (*y*‐axis). The bubble size is proportional to the number of observations. Effects of treatment categories were classified (horizontal panels) as “positive” (green bubbles), “variable” (blue bubbles), “not significant” (gray bubbles), or “negative” (orange bubbles).

Nitrogen addition was the most common treatment, comprising 9.24% of all observations (*n* = 103 of 1114) and 55.38% of fertilization studies (*n* = 103 of 186) across 16 studies and 12 papers (Figure 4). This treatment showed a predominantly nonsignificant effect (51.5%). The rest of the observations were mainly positive (20.4% of the total observations; *n* = 53, 21, 15 and 14 observations with nonsignificant, positive, variable and negative effects, respectively). These positive effects of nitrogen fertilization align with the understanding that tree growth is frequently constrained by nitrogen availability (Rennenberg et al. 1998; Nadelhoffer 2000; Pretzsch et al. 2014). However, the presence of negative effects of nitrogen fertilization indicates that nitrogen addition does not universally enhance seedling recruitment in temperate and subtropical trees. Adverse effects may arise from nutrient imbalances, soil acidification, or alterations in microbial and mycorrhizal communities that constrain seedling performance under certain conditions (Bobbink et al. 2010). In addition, variation among experiments may partly explain contrasting outcomes, as these may depend on fertilizer dose and background seedbed fertility (Bobbink et al. 2010).

### 4.2 Abiotic Climatic Modifiers—Limited Temperature and Precipitation Effects Compared to More Consistent Soil Moisture and Snow Cover Effects on Seedlings

Two categories of abiotic climatic modifiers, temperature and temperature regimes, were most prevalent across our reviewed papers (Figure 4, Table 3). Nearly half of the observations (46.9%) of the effects of temperature and temperature regime indicated nonsignificant effects (*n* = 53 of 113; for temperature: *n* = 25, 13, 9, and 5 observations with nonsignificant, negative, positive and variable effects, respectively; for temperature regime: *n* = 28, 20, and 13 observations with nonsignificant, negative and variable effects, respectively). Among the significant effects (*n* = 60), increased temperature and temperature‐regime modifications had often negative (*n* = 32) and variable (*n* = 18) effects on seed germination and germination time (n.b. negative effects on germination time mean a reduction of the germination time), seedling survival and growth of temperate and subtropical trees. Effects of precipitation were also largely nonsignificant (*n* = 15 of 29), particularly on seedling survival (*n* = 8 of 15).

**TABLE 3:** Treatment categories of abiotic climatic modifiers with their respective number of observations, studies, and papers.

Treatment categories of abiotic climatic modifiers	No. observations	No. studies	No. papers
Temperature regime	61	5	5
Temperature	52	11	11
Precipitation	29	3	3
Latitude	20	1	1
Soil moisture	20	3	2
Snow cover	19	2	2
Flooding	6	1	1
Temperature & precipitation	6	1	1
Drought	2	1	1
Seasonality	2	2	1
Elevation	1	1	1
Total	218	31	29

The large proportion of nonsignificant effects found contrasts with the expectation that rising temperatures and increased drought, as resulting from climate change, diminish seedling recruitment (Niinemets 2010). This expectation is based on the assumption that higher temperatures elevate evaporation and water stress, thereby increasing mortality risk and reducing establishment success in early life stages. Against this backdrop, the absence of consistent negative effects is notable. However, this pattern is unlikely to be an artifact of limited statistical power, as the studies examining these drivers were generally well‐designed. Instead, the prevalence of nonsignificant results may reflect the capacity of many temperate tree species to persist across a wide range of climatic conditions, as indicated by their broad geographic distributions (Savolainen et al. 2007). Climatic variations within a tree's tolerable range may trigger phenotypic plasticity (Richter et al. 2012), and thus may not have a noticeable short‐term effect on the response variables we reviewed. This would be rather promising, as phenotypic plasticity is believed to be crucial for the survival of tree species—particularly slow‐growing ones—under climate change (Grulke 2010; Vitasse et al. 2010).

In contrast to the preceding patterns, soil moisture and snow cover had relatively consistent positive effects on seedling recruitment (for soil moisture: *n* = 9, 2, 9 observations with nonsignificant, negative and positive effects, respectively; for snow cover: *n* = 7, 2, 10 observations with nonsignificant, negative and positive effects, respectively; Figure 4). These effects likely reflect reduced drought stress and enhanced protection from (frost) desiccation, supporting the view that water availability is a critical factor for seedling survival, particularly in harsh environments such as treeline ecotones (Körner 2012). Nonetheless, these findings contrast with the expectation of more uniformly positive and fewer nonsignificant effects of moisture across the various seedling‐recruitment metrics evaluated for temperate and subtropical tree species. One possible explanation is that moisture may only enhance recruitment when it alleviates limiting conditions, whereas under already mesic conditions its additional effects remain marginal and therefore statistically undetectable.

### 4.3 Abiotic Environmental Modifiers—Shading Reduces and Soil Scarification Enhances Seedlings, While Evidence for Pollution and Light Is Lacking

Among the abiotic environmental modifiers, shading was the most studied modifier, followed by pollution, light regime, light availability and soil scarification (Figure 4; Table 4). Effects of shading were generally detrimental to seed germination, seedling survival, and growth, with more than half of the observations reporting negative effects (*n* = 101 of 188; *n* = 101, 56, 33 and 2 observations had negative, nonsignificant, positive and variable effects, respectively). The predominantly negative effects of shading likely reflect light limitation during early recruitment stages, as reduced irradiance constrains photosynthetic carbon gain and can shift biomass allocation patterns, particularly in light‐demanding temperate species (e.g., Lu et al. 2018). Comparisons among the reviewed experiments further suggest that responses to shading depended on the species' shade‐tolerance at the seedling stage and that moderate canopy cover can buffer seedlings against environmental stress, highlighting the context dependency of light effects across site conditions and species functional types (e.g., Lu et al. 2018; McAlpine and Drake 2003). Salinity had consistent, negative effects across all observations (*n* = 6), induced by osmotic stress and ion toxicity mechanisms, which impair water uptake, during germination and early seedling development (e.g., Aljasmi et al. 2021). In contrast, the generally positive responses to soil scarification (*n* = 8 of 11, the three other observations were nonsignificant), a treatment disturbing the litter layer and understory vegetation to increase the availability of mineral soil and humus (Willis et al. 2015), likely result from enhanced access to mineral soil, and reduced competition from understory vegetation, with some experiments reporting particularly strong benefits in small‐seeded species (Willis et al. 2015; Clark and D'Amato 2023).

**TABLE 4:** Treatment categories of abiotic environmental modifiers with their respective number of observations, studies, and papers.

Treatment categories of abiotic environmental modifiers	No. observations	No. studies	No. papers
Shading	192	29	23
Pollution	65	6	6
Light regime	24	2	2
Light	12	4	4
Soil scarification	11	2	2
Forest‐gap size	6	1	1
Salinity	6	2	2
Wind protection	6	2	1
Distance to seashore	4	2	2
Plantation date	1	1	1
Time since plantation	1	1	1
Total	326	43	39

Pollution, including acid rain, extreme increase of nitrogen deposition, CO_2_ and ozone concentration (Percy 1986; McConnaughay et al. 1996; Hättenschwiler and Körner 2003; Niu et al. 2011; BassiriRad et al. 2015), had predominantly nonsignificant effects on seed germination and seedling growth and survival (*n* = 40 out of 65). Similarly, effects of light availability (Aljasmi et al. 2021; Galíndez et al. 2019; Hadi et al. 2018; Velazco et al. 2018) and light regime, such as red‐light pulses and increased far‐red light proportion (Lacoretz et al. 2022; Xia et al. 2016), were reported to be mainly nonsignificant on seed germination and germination time (for light‐increase effects: *n* = 8 nonsignificant observations of 11; for light‐regime effects: *n* = 14 nonsignificant observations of 24).

### 4.4 Mixed Biotic and Abiotic Modifiers – Inconclusive Effects and Lack of Evidence on Seedling Recruitment

We defined mixed biotic and abiotic modifiers as any modifier of seed and/or seedling that implies both biotic and abiotic processes (Table 2). Among these modifiers, litter treatments, encompassing litter addition and litter‐depth increase (Table 1), received the most extensive scrutiny (*n* = 59 of 122), with 10 studies across eight papers. On seed germination and seedling growth, this treatment category mainly indicated nonsignificant and positive effects (35.2% and 31.5% of the observations, respectively; *n* = 8, 6, 3, and 2 observations with positive, nonsignificant, negative and variable effects on germination; *n* = 13, 9, 9, and 4 observations with nonsignificant, positive, variable and negative effects on growth; Figure 4). On seedling survival and germination time, no tendency in the effect of litter treatments could be identified due to data scarcity (for survival: *n* = 4 observations with mixed results; for germination time: *n* = 1 observation). Research has demonstrated that plant litter can positively influence tree seedling establishment by creating favorable microhabitats through humidity and nutrient availability enhancement, and light and soil temperature modification (Vázquez‐Yanes and Orozco‐Segovia 1992; Facelli and Pickett 1991; Martínez‐Garza et al. 2005; Dupuy and Chazdon 2008; Urretavizcaya and Defossé 2013; Bürli et al. 2026).

Conversely, litter addition can also alter negatively tree seedling recruitment through allelopathy (chemically mediated interference between co‐occurring species; Muller 1965; Sujeeun and Thomas 2023). In our review, however, there were only six observations (from two studies) on the effects of allelopathic molecules alone (and not on the effects of litter addition), which showed no clear pattern in relation to tree seedling recruitment (with two observations each reporting nonsignificant, negative, and variable effects). Lastly, we identified a significant data gap regarding the effects of nurse species, forest canopy age, soil (origin, type, and sterilization), and understory treatments on the early life stages of temperate and subtropical trees, with four or fewer studies investigating each of these factors. Given the importance of these modifiers for ecosystem restoration efforts (e.g., Padilla and Pugnaire 2006), further research in these areas could lead to the development of more effective restoration strategies.

### 4.5 Biotic Modifiers—Mycorrhizae Promote, While Competition Had Variable Effects on Seedling Recruitment

Mycorrhizae received the most attention among the seven biotic modifiers and their effects were examined on seedling growth and survival (*n* = 30 and 11; Figure 4). Although most observations indicated positive effects of mycorrhizae (*n* = 17 of 41), several nonsignificant (*n* = 12), and variable (*n* = 12) effects were also reported. Mycorrhizae are widely recognized for their importance in enhancing nutrient uptake and overall plant health (Martin et al. 2017). However, studying their effects on seedling growth and survival is complex and this stems from multiple interacting variables: Firstly, the impact of mycorrhizae can be influenced by soil type and nutrient availability. For example, in nutrient‐rich soils, the benefits of mycorrhizal association may be less pronounced compared to nutrient‐poor environments where these fungi can play a crucial role in facilitating nutrient uptake (Hoeksema et al. 2010). Secondly, different plant species exhibit varying degrees of dependency on mycorrhizal relationships. Some species may benefit significantly from these associations, while others may show minimal response (Gerdemann et al. 1975; Siqueira and Saggin‐Júnior 2001). Lastly, the effects of mycorrhizae can also vary depending on the specific types of mycorrhizae involved (e.g., arbuscular, ectomycorrhizal). Given that each type differs in their ecophysiology, effects on soil and interactions with host plants (Tedersoo and Bahram 2019), diverse outcomes on seedling growth and survival is not surprising. Hence, the mixed mycorrhizal effects we found reflect the complexity of such experiments and highlight the need for further research to determine whether consistent patterns exist in the mycorrhizal effects on early establishment of temperate and subtropical tree species. To draw generalizable conclusions across species and ecosystems, broader experiments encompassing diverse plant traits, species, and habitats are essential.

Competition had mainly negative and nonsignificant effects reported on seedling growth and survival (on growth: *n* = 8, 7, and 6 observations with nonsignificant, negative and positive effects, respectively; on survival: *n* = 3 and 1 observations with nonsignificant and negative effects, respectively). However, six observations from two studies indicated positive effects of competition on seedling growth: Carswell et al. (2012) used root trenching to reduce belowground competition and found that it negatively affected seedling diameter (*n* = 3) but positively affected height (*n* = 3) in three species. Yin et al. (2023) observed contrasting responses of seedling density to conspecific seedling density: *Tilia amurensis* showed a positive response, while *Fraxinus mandshurica* responded negatively—likely due to differences in shade tolerance, biomass allocation, and mycorrhizal type. These findings highlight the complex and species‐specific effects of competition on seedling recruitment in temperate and subtropical trees. Such variability likely reflects differences in life‐history strategies and ecological traits (e.g., competitive ability; Grime 1979), as well as unintended side effects of experimental methods. For example, root trenching may alter soil moisture (Carswell et al. 2012), confounding competition effects. Future studies should aim to minimize such confounding factors through careful experimental design.

## 5 What Seed Priming and Seedling Treatment Techniques Were Most Prevalent and What Were Their Effects on Seedling Recruitment‐Parameters?

### 5.1 Seed Priming—Seed Stratification Improved Germination More Effectively Than Seed Scarification and Chemical Treatments

**FIGURE 5:**
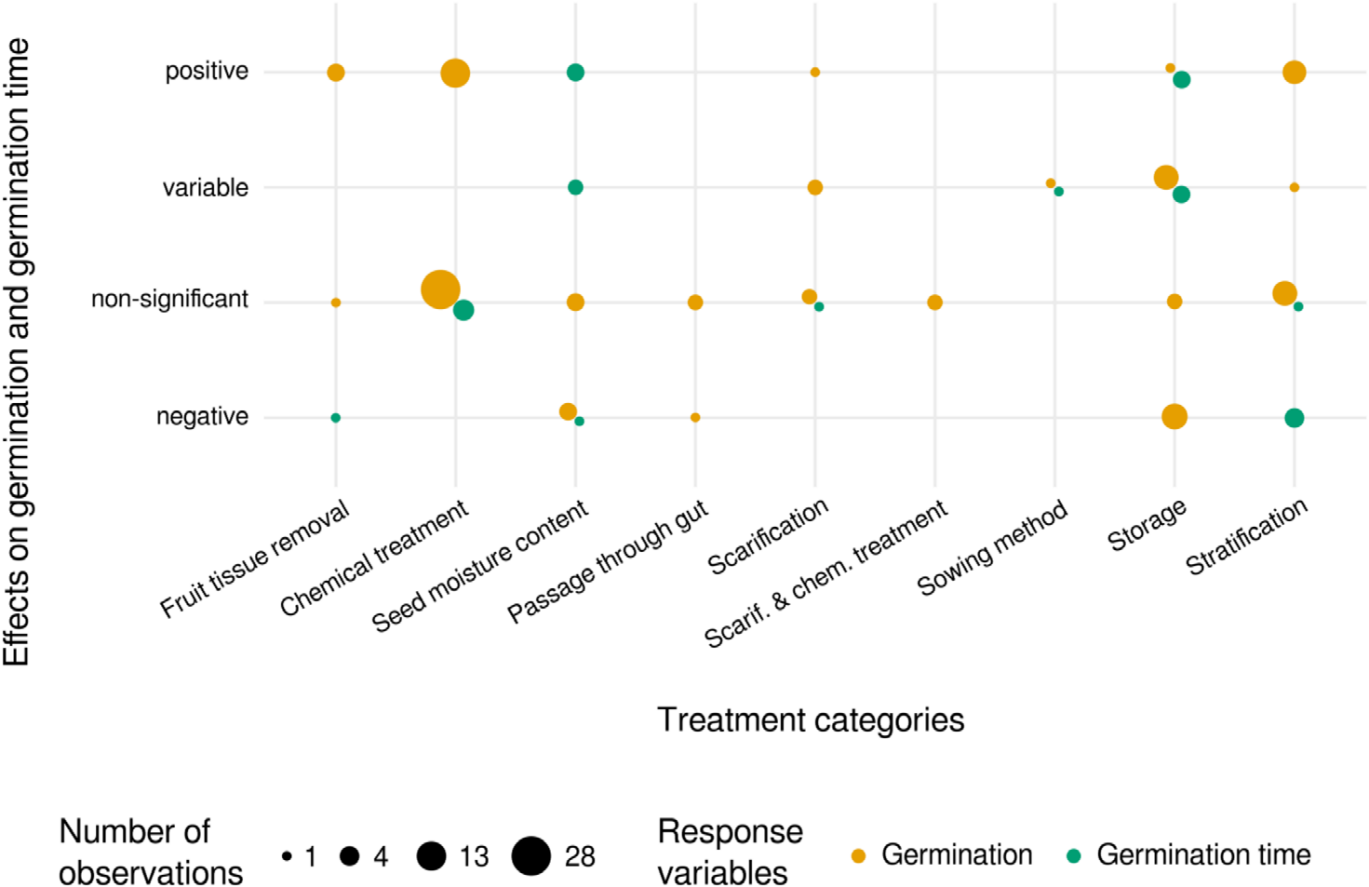
Cross‐tabulation results for the 20 reviewed studies (across 18 papers) that investigated effects of nine treatment categories (*x*‐axis) tested on seed germination (orange bubbles) and germination time (green bubbles). The bubble size is proportional to the number of observations. Effects of treatment categories (*y*‐axis) were classified as “positive,” “variable,” “not significant,” or “negative.” “Passage through gut” refers to the seed treatment involving their passage through an animal's gut. “Scarif. & chem. treatment” stands for scarification and chemical treatment.

Nine categories of seed priming techniques were investigated on seed germination (20 studies from 18 papers) and seven of them were also investigated on germination time (six studies from five papers; Figure 5). Most observations (*n* = 94 of 124) were reported for three technique categories: seed chemical treatment, storage time and condition, and stratification.

Our review highlighted that seed stratification improved germination (*n* = 7 and 1 observations with positive and variable effects, respectively) and decreased germination time (*n* = 4 and 1 observations with negative and nonsignificant effects, respectively) in most observations. Stratification is a common seed priming technique used to break dormancy and promote germination in plant propagation from seeds, particularly in botanical and horticultural institutions (personal observations). Among the reviewed studies, stratification temperatures ranged from 4°C to 40°C. Species with physiological dormancy (i.e., species needing physiological changes for radicle emergence; Geneve 2003), notably those from high elevations and latitudes, typically require cold, moist conditions to break dormancy and initiate germination (Milbau et al. 2009; Körner 2013). In contrast, some species require warm stratification (temperatures > 15°C) to germinate (e.g., Baskin et al. 2002; Geneve 2003). While most studies we reviewed focused on cold stratification, only three experiments across two papers investigated warm stratification. This highlights that gaining insights into the effects of warm stratification is crucial for developing a comprehensive understanding of the stratification efficiency on tree species across the temperate and subtropical biomes.

Seed scarification, whether it is done chemically or mechanically, is another widely used practice to increase seed germination. It is recommended for species having a physical dormancy (i.e., seeds with anatomical structures preventing water uptake), such as species from the Fabaceae, Malvaceae, Cannaceae, Convolvulaceae and Elaeocarpaceae families (Baskin et al. 2000; Iralu et al. 2019). In the reviewed studies, scarification was however tested predominantly on other plant families. The variability in scarification effects we found (*n* = 2, 2, and 1 observations with nonsignificant, variable, and positive effects on germination, respectively) suggests its efficacy depends widely on species. Given its common use in seed priming (Baskin et al. 2000), better understanding of its effects would enhance seed propagation efforts.

Although seed chemical treatments were the seed priming category that attracted the most interest, most observations (80.2%) indicated nonsignificant effects on seed germination and germination time (*n* = 33, 13 and 1 observations with nonsignificant, positive, and negative effects). We therefore recommend conducting further investigations to determine the conditions under which chemical treatments affect seed germination in plants from temperate and subtropical biomes. According to the current knowledge highlighted by our review, ecologists and plant practitioners may consider stratifying the seeds as an alternative seed priming technique to chemical treatment to enhance seed germination.

Seed storage prior to germination and high seed moisture level mainly decreased or had variable effects on seed germination (for seed storage: *n* = 9, 8, 2, and 1 observations with negative, variable, nonsignificant and positive effects, respectively; for seed moisture level: *n =* 3 and 3 observations with negative and nonsignificant effects, respectively). On germination time, seed storage had mixed results (for seed storage: *n =* 3 and 3 observations out of 6 with positive and variable effects, respectively; for seed moisture level: *n =* 3, 2, and 1 observation(s) out of 6 with positive, variable and negative effects, respectively). Aging pace has been shown to be significantly influenced by environmental and genetic factors, including storage temperature, seed moisture content, and overall seed quality (Walters 1998; Walters et al. 2005; Rajjou and Debeaujon 2008; Iralu et al. 2019). Our findings align with the understanding that prolonged seed storage or high seed moisture content accelerates seed aging, reducing germination.

### 5.2 Seedling Treatments—Conclusions Hindered by Research Scarcity

Compared to seed priming techniques, our review found that seedling treatments have received relatively little research attention regarding their effects on seedling survival and growth. Effects of only four categories of seedling treatments were investigated: pot size in which seedlings were grown (McConnaughay et al. 1996), pot coating with copper carbonate (CuCO_3_; Dunn et al. 1997), seedling transplantation method (Velazco et al. 2018), and seedling size (Osunkoya and Creese 1997). Moreover, four studies in total, each one from a different paper, investigated the effects of these four seedling treatments on only two seedling traits: growth and survival. The limited number of reported observations on the effects of seedling treatments prevents us from drawing any conclusions about their impact on seedlings.

## 6 What Knowledge Gaps Remain, and What Directions Should Future Research Take?

Across the body of literature reviewed, nonsignificant effects of modifiers and treatments were frequently reported across the seed and seedling response‐categories investigated. We identified two potential causes of this outcome. First, the experimental designs of the reviewed studies may not have met the necessary requirements for adequate statistical power, and sample sizes might have been insufficient to detect significant effects (higher type II error rate due to low power). However, we consider this an unlikely primary cause, as we excluded studies with obviously inadequate sample sizes. It is possible, nonetheless, that there may still be a discrepancy between ecologically reasonable sample sizes, shaped by logistical constraints, such as seed collection, species diversity, and study complexity, and the sample sizes required for statistical robustness. Second, and perhaps most importantly, the inherently variable nature of ecological systems may result in a higher proportion of nonsignificant outcomes than typically expected. This contrasts with the prevailing assumption that modifiers will consistently yield measurable effects.

In addition to a high prevalence of nonsignificant effects, we observed substantial heterogeneity in the effects of modifiers and treatments on the seed and seedling response‐categories investigated. The strong context dependence of ecological processes (Catford et al. 2022; Spake et al. 2023) likely contributes to the heterogeneity observed within and across studies. This variability arises because ecological processes, such as seedling recruitment, are influenced by numerous local factors (e.g., soil type, microclimate, biotic interactions; Das and Biswas 2022), which may not be consistently accounted for in studies. As a result, this complicates efforts to identify clear and generalizable patterns in the effects of modifiers and treatments. Taken together, the high proportion of nonsignificant results and the strong heterogeneity across studies point to a fundamental gap in our mechanistic understanding of the effects of biotic and abiotic modifiers and treatment on seedling recruitment. To fill this gap, we need to clarify how contextual factors shape ecological outcomes and identify the key drivers of variation in seedling recruitment. This requires to reassess ecological hypotheses and improve experimental designs to better capture the processes influencing seedling recruitment and incorporate context‐specific variables.

Beyond the heterogenous and nonsignificant effects, our systematic narrative review reveals substantial and systematic research gaps that constrain progress in understanding seedling recruitment‐dynamics. Several modifiers and experimental treatments remain markedly underrepresented in the literature. For instance, studies on liming and total soil amendment treatments, such as ashes, biochar and charcoal addition, are scarce, despite their increasing relevance in restoration and soil management. Likewise, several abiotic climatic modifiers, e.g., drought, flooding, elevation, latitude, and seasonality, and non‐climatic environmental modifiers, e.g., forest gap size, wind protection, distance to seashore, plantation date, and time since plantation, have been rarely investigated. Key biotic modifiers also remain largely unstudied. In particular, the effects of nitrogen‐fixing bacteria, antagonistic interactions with herbivores and pathogens, species richness, and animal introductions are poorly understood in the context of seedling recruitment. Similarly, seed priming techniques, including fruit tissue removal, seed passage through animal gut, combined scarification and chemical treatments, and sowing methods, and experimental seedling treatments are under‐explored. This research scarcity limits our ability to evaluate their effectiveness and to compare their outcomes across species from temperate and subtropical biomes.

With an average annual increase of approximately 6% (266 records identified on 15th August 2023 using our search query versus 307 on 18th February 2026), substantial time will be required before a sufficiently large and coherent body of evidence accumulates to support clear conclusions regarding the effects of the examined modifiers and treatments. To accelerate the advancement of our knowledge, future research should prioritize: (i) systematically testing under‐represented abiotic and biotic modifiers across environmental gradients. In this context, the following specific knowledge gaps have been highlighted: the role of mycorrhizal associations in seedling recruitment across diverse ecosystems, effects of fertilization across doses and species, stratification (particularly warm stratification), scarification and chemical treatments to evaluate their relative effectiveness in enhancing germination and seedling performance across a wide range of species, and experiments on interspecific and intraspecific competition with careful attention to controlling potential confounding factors. (ii) Future studies should explicitly incorporate context‐specific variables to capture the influence of local environmental conditions on modifier and treatment effects. (iii) Research should adopt experimental designs that facilitate cross‐study synthesis and improve the generalizability of findings.

Given that plant functional traits shape species' responses to both natural modifiers and experimental treatments, a trait‐based research approach holds promise for filling these knowledge gaps. Linking modifier and treatment effects to life‐history strategies and functional traits could help identify generalizable patterns and improve predictive capacity in ecology and conservation (Loureiro et al. 2023). By integrating trait‐based approaches with context‐explicit experimental designs, future studies can move beyond isolated findings to achieve a more mechanistic and generalizable understanding of seedling recruitment. Advancing our knowledge of how modifiers and treatments influence seedling recruitment will ultimately improve seedling establishment and propagation in restoration contexts, which are essential for mitigating the impacts of climate change on biodiversity.

## Author Contributions

**Sarah Bürli:** conceptualization (equal), data curation (lead), formal analysis (lead), investigation (lead), methodology (equal), project administration (equal), visualization (lead), writing – original draft (lead), writing – review and editing (lead). **Hannah L. Buckley:** conceptualization (equal), data curation (equal), formal analysis (equal), funding acquisition (equal), investigation (equal), methodology (equal), project administration (equal), visualization (equal), writing – original draft (equal), writing – review and editing (equal). **Bradley S. Case:** conceptualization (equal), data curation (equal), formal analysis (equal), funding acquisition (equal), investigation (equal), methodology (equal), project administration (equal), visualization (equal), writing – original draft (equal), writing – review and editing (equal).

## Funding

This work was supported by grants from the New Zealand Forest Service|Te Uru Rākau (Contract No. 1BT‐01971‐AUT) and from New Zealand's Biological Heritage National Science Challenge (Contract No. 2223–44‐009).

## Conflicts of Interest

The authors declare no conflicts of interest.

## Supporting information


**Material S1.** Search string for literature search in Scopus database done on the 15 August 2024.
**Material S2**. List of the 266 papers identified by our search string from the Scopus database (https://www.scopus.com) on the 15 August 2023.

## Data Availability

Data are archived on the international open‐access repository DRYAD, https://doi.org/10.5061/dryad.j0zpc86wf.
