# Supplementary material for: Tree Seedling‐Recruitment in Temperate and Subtropical Species: A Systematic Narrative Review of Biotic and Abiotic Modifiers, and Experimental Treatments
Source: Ecol Evol. 2026 Apr 10;16(4):e73399. doi: 10.1002/ece3.73399 (PMC13066763; doi:10.1002/ece3.73399)
Supplement: Supplementary file 1 — Material S1. Search string for literature search in Scopus database done on the 15 August 2024. Material S2. List of the 266 papers identified by our search string from the Scopus database (https://www.scopus.com) on the 15 August 2023. [file ECE3-16-e73399-s001.docx]

**Tree Seedling-Recruitment in Temperate and Subtropical Species: A Systematic Narrative Review of Biotic and Abiotic Modifiers, and Experimental Treatments**

**Bürli, S., Buckley, H. L., & Case, B. S. (2026; published in Ecology and Evolution Journal)**

# SUPPORTING MATERIAL S1

*Search string for literature search in Scopus database done on the 15 August 2024. ABS denotes “abstract” and indicates that the words were looked for in the paper abstracts. We included the terms "New Zealand" and "Aotearoa" in our search query to conduct a sub-study required by our funding institution, which focused on examining the impact of biotic and abiotic factors and experimental treatments on the seedlings recruitment of tree species from Aotearoa New Zealand. Incorporating these two terms yielded 25 additional papers compared to the search query that did not include them.*

ABS ((tree* OR conifer* OR broad-lea*) AND (“seed germination” OR “seed dormancy” OR “seedling establishment” OR “seedling performance” OR “seedling growth” OR “seedling fitness” OR “seedling survival” OR “seedling mortality” OR “germinant establishment” OR “germinant performance” OR “germinant growth” OR “germinant fitness” OR “germinant survival” OR “germinant mortality” OR “juvenile establishment” OR “juvenile performance” OR “juvenile growth” OR “juvenile fitness” OR “juvenile survival” OR “juvenile mortality”) AND (treatment* OR experiment* OR effect* OR respon* OR correla* OR trial* OR impact* OR techn* OR factor* OR role* OR tempera* OR hydra*) AND (temperate* OR subtropic* OR "New Zealand" OR aotearoa) AND NOT (herb* OR forb* OR graminoid* OR sedge* OR grass* OR cushion* OR tropic* OR alpin* OR nival* OR boreal* OR antarctic* OR arctic* OR tundra OR mountain* OR montan* OR snowbed* OR crop*) )

# SUPPORTING MATERIAL S2

*List of the 266 papers identified by our search string from the Scopus database (https://www.scopus.com) on the 15 August 2023.*

Abe, S., T. Nakashizuka, and H. Tanaka. ‘Effects of Canopy Gaps on the Demography of the Subcanopy Tree Styrax Obassia’. *Journal of Vegetation Science* 9, no. 6 (1998): 787–96. <https://doi.org/10.2307/3237044>.

Abe, T. ‘Effects of Treeshelter on Seedling Performance: A Meta-Analysis’. *Journal of Forest Research* 27, no. 3 (2022): 171–81. <https://doi.org/10.1080/13416979.2021.1992700>.

Ahmadi, E., S.M.H. Nasr, H. Jalilvand, and S.K. Savadkoohi. ‘Contamination Control of Microbe Ziziphus Spina [Christti] Seed in Vitro Culture’. *Trees - Structure and Function* 26, no. 4 (2012): 1299–1304. <https://doi.org/10.1007/s00468-012-0705-8>.

Ajamgard, F. ‘Selection of Pecan Cultivars Aiming to Release Vigorous and Heat Stress Tolerant Rootstocks’. *Journal of Nuts* 13, no. 1 (2022): 57–70. <https://doi.org/10.22034/jon.2022.1942678.1139>.

Akaji, Y., M. Hirobe, Y. Miyazaki, T. Makimoto, S. Kinoshita, I. Hattori, and K. Sakamoto. ‘Survival and Growth of Fagus Crenata Seedlings in Relation to Biological and Microtopographical Factors in a Cool Temperate Broadleaf Forest’. *Journal of Forest Research* 22, no. 5 (2017): 294–302. <https://doi.org/10.1080/13416979.2017.1354749>.

Akaji, Y., Y. Miyazaki, M. Hirobe, T. Makimoto, and K. Sakamoto. ‘The Relationship between Seedling Survival Rates and Their Genetic Relatedness to Neighboring Conspecific Adults’. *Plant Ecology* 217, no. 4 (2016): 465–70. <https://doi.org/10.1007/s11258-016-0591-z>.

Aljasmi, M., A. El-Keblawy, and K.A. Mosa. ‘Abiotic Factors Controlling Germination of the Multipurpose Invasive Prosopis Pallida: Towards Afforestation of Salt-Affected Lands in the Subtropical Arid Arabian Desert’. *Tropical Ecology* 62, no. 1 (2021): 116–25. <https://doi.org/10.1007/s42965-020-00124-3>.

Arseneau, J., N. Bélanger, R. Ouimet, S. Royer-Tardif, S. Bilodeau-Gauthier, B. Gendreau-Berthiaume, and D. Rivest. ‘Wood Ash Application in Sugar Maple Stands Rapidly Improves Nutritional Status and Growth at Various Developmental Stages’. *Forest Ecology and Management* 489 (2021). <https://doi.org/10.1016/j.foreco.2021.119062>.

Ashworth, L., A. Calviño, M.L. Martí, and R. Aguilar. ‘Offspring Performance and Recruitment of the Pioneer Tree Acacia Caven (Fabaceae) in a Fragmented Subtropical Dry Forest’. *Austral Ecology* 40, no. 6 (2015): 634–41. <https://doi.org/10.1111/aec.12230>.

Bang-Xing, W. ‘Study on the Dynamics and Rhythms of Midmontane Wet Evergreen Broad- Leaved Forest at Xujiaba, Ailao Mountains, Yunnan’. *Acta Botanica Sinica* 37, no. 12 (1995): 869–977.

Bai, X., S.A. Queenborough, X. Wang, J. Zhang, B. Li, Z. Yuan, D. Xing, F. Lin, J. Ye, and Z. Hao. ‘Effects of Local Biotic Neighbors and Habitat Heterogeneity on Tree and Shrub Seedling Survival in an Old-Growth Temperate Forest’. *Oecologia* 170, no. 3 (2012): 755–65. <https://doi.org/10.1007/s00442-012-2348-2>.

BassiriRad, H., J.F. Lussenhop, H.L. Sehtiya, and K.K. Borden. ‘Nitrogen Deposition Potentially Contributes to Oak Regeneration Failure in the Midwestern Temperate Forests of the USA’. *Oecologia* 177, no. 1 (2015): 53–63. <https://doi.org/10.1007/s00442-014-3119-z>.

Bayandala, Y.F., and K. Seiwa. ‘Roles of Pathogens on Replacement of Tree Seedlings in Heterogeneous Light Environments in a Temperate Forest: A Reciprocal Seed Sowing Experiment’. *Journal of Ecology* 104, no. 3 (2016): 765–72. <https://doi.org/10.1111/1365-2745.12552>.

Bendall, E.R., M. Bedward, M. Boer, H. Clarke, L. Collins, A. Leigh, and R.A. Bradstock. ‘Changes in the Resilience of Resprouting Juvenile Tree Populations in Temperate Forests Due to Coupled Severe Drought and Fire’. *Plant Ecology* 223, no. 7 (2022): 907–23. <https://doi.org/10.1007/s11258-022-01249-2>.

Bettoni, J.C., K. van der Walt, J.A. Souza, A. McLachlan, and J. Nadarajan. ‘Sexual and Asexual Propagation of Syzygium Maire, a Critically Endangered Myrtaceae Species of New Zealand’. *New Zealand Journal of Botany*, 2022. <https://doi.org/10.1080/0028825X.2022.2158110>.

Birge, Z.K.D., K.F. Salifu, and D.F. Jacobs. ‘Modified Exponential Nitrogen Loading to Promote Morphological Quality and Nutrient Storage of Bareroot-Cultured Quercus Rubra and Quercus Alba Seedlings’. *Scandinavian Journal of Forest Research* 21, no. 4 (2006): 306–16. <https://doi.org/10.1080/02827580600761611>.

Bohora Schlickmann, M., A.C. da Silva, L.M. de Oliveira, D. Oliveira Matteucci, F. Domingos Machado, T. Cuchi, E. Duarte, and P. Higuchi. ‘Specific Leaf Area Is a Potential Indicator of Tree Species Sensitive to Future Climate Change in the Mixed Subtropical Forests of Southern Brazil’. *Ecological Indicators* 116 (2020). <https://doi.org/10.1016/j.ecolind.2020.106477>.

Borges, R.G., and W.R. Chaney. ‘The Response of Acacia Scleroxyla Tuss. to Mycorrhizal Inoculation’. *International Tree Crops Journal* 5, no. 3 (1988): 191–201. <https://doi.org/10.1080/01435698.1988.9752853>.

Boudreau, S., and M.J. Lawes. ‘Small Understorey Gaps Created by Subsistence Harvesters Do Not Adversely Affect the Maintenance of Tree Diversity in a Sub-Tropical Forest’. *Biological Conservation* 126, no. 2 (2005): 279–86. <https://doi.org/10.1016/j.biocon.2005.06.004>.

Brown, S.L., J. Reid, N. Reid, and R. Smith. ‘Differences in Tree and Shrub Establishment Due to Tree Guard Type in a Temperate Upland Pasture’. *Ecological Management and Restoration* 19, no. 2 (2018): 166–68. <https://doi.org/10.1111/emr.12304>.

Burrows, C.J. ‘Forest Regeneration Patterns in New Zealand’s Turbulent Environments’. *Polish Botanical Studies* 22 (2006): 95–122.

Burrows, L., E. Cieraad, and N. Head. ‘Scotch Broom Facilitates Indigenous Tree and Shrub Germination and Establishment in Dryland New Zealand’. *New Zealand Journal of Ecology* 39, no. 1 (2014): 61–70.

Campbell, D.J. ‘Salt-Wind Induced Wave Regeneration in Coastal Pine Forests in New Zealand’. *Canadian Journal of Forest Research* 28, no. 7 (1998): 953–60. <https://doi.org/10.1139/x98-073>.

Campbell, D.J., and I.A.E. Atkinson. ‘Effects of Kiore (Rattus Exulans Peale) on Recruitment of Indigenous Coastal Trees on Northern Offshore Islands of New Zealand’. *Journal of the Royal Society of New Zealand* 29, no. 4 (1999): 265–90. <https://doi.org/10.1080/03014223.1999.9517597>.

Candaele, R., G. Ligot, A. Licoppe, J. Lievens, V. Fichefet, M. Jonard, F. André, and P. Lejeune. ‘Interspecific Growth Reductions Caused by Wild Ungulates on Tree Seedlings and Their Implications for Temperate Quercus-Fagus Forests’. *Forests* 14, no. 7 (2023). <https://doi.org/10.3390/f14071330>.

Carón, M.M., P. De Frenne, J. Brunet, O. Chabrerie, S.A.O. Cousins, L. De Backer, G. Decocq, et al. ‘Interacting Effects of Warming and Drought on Regeneration and Early Growth of Acer Pseudoplatanus and A. Platanoides’. *Plant Biology* 17, no. 1 (2015): 52–62. <https://doi.org/10.1111/plb.12177>.

Carón, M.M., P. De Frenne, P. Ortega-Baes, A. Quinteros, and K. Verheyen. ‘Regeneration Responses to Climate and Land-Use Change of Four Subtropical Tree Species of the Southern Central Andes’. *Forest Ecology and Management* 417 (2018): 110–21. <https://doi.org/10.1016/j.foreco.2018.02.006>.

Carrari, E., E. Ampoorter, F. Bussotti, A. Coppi, A. Garcia Nogales, M. Pollastrini, K. Verheyen, and F. Selvi. ‘Effects of Charcoal Hearth Soil on Forest Regeneration: Evidence from a Two-Year Experiment on Tree Seedlings’. *Forest Ecology and Management* 427 (2018): 37–44. <https://doi.org/10.1016/j.foreco.2018.05.038>.

Carswell, F.E., J.E. Doherty, R.B. Allen, M.E. Brignall-Theyer, S.J. Richardson, and S.K. Wiser. ‘Quantification of the Effects of Aboveground and Belowground Competition on Growth of Seedlings in a Conifer-Angiosperm Forest’. *Forest Ecology and Management* 269 (2012): 188–96. <https://doi.org/10.1016/j.foreco.2011.12.027>.

Catovsky, S., and F.A. Bazzaz. ‘Feedbacks between Canopy Composition and Seedling Regeneration in Mixed Conifer Broad-Leaved Forests’. *Oikos* 98, no. 3 (2002): 403–20. <https://doi.org/10.1034/j.1600-0706.2002.980305.x>.

Catovsky, S., and F.A. Bazzaz.‘Nitrogen Availability Influences Regeneration of Temperate Tree Species in the Understory Seedling Bank’. *Ecological Applications* 12, no. 4 (2002): 1056–70. [https://doi.org/10.1890/1051-0761(2002)012[1056:NAIROT]2.0.CO;2](https://doi.org/10.1890/1051-0761(2002)012%5B1056:NAIROT%5D2.0.CO;2).

Catovsky, S., R.K. Kobe, and F.A. Bazzaz. ‘Nitrogen-Induced Changes in Seedling Regeneration and Dynamics of Mixed Conifer-Broad-Leaved Forests’. *Ecological Applications* 12, no. 6 (2002): 1611–25. [https://doi.org/10.1890/1051-0761(2002)012[1611:NICISR]2.0.CO;2](https://doi.org/10.1890/1051-0761(2002)012%5B1611:NICISR%5D2.0.CO;2).

Cavallero, L., and M. Blackhall. ‘Resprouting Increases Seedling Persistence Likelihood after Fire in a Semelparous Bamboo Species’. *Acta Oecologica* 108 (2020). <https://doi.org/10.1016/j.actao.2020.103623>.

Chen, H., W. Quan, H. Liu, and G. Ding. ‘Effects of Suillus Luteus and S. Bovinus on the Physiological Response and Nutrient Absorption of Pinus Massoniana Seedlings under Phosphorus Deficiency’. *Plant and Soil* 471, no. 1–2 (2022): 577–90. <https://doi.org/10.1007/s11104-021-05211-5>.

Chmura, D.J., J. Modrzyński, P. Chmielarz, and M.G. Tjoelker. ‘Plasticity in Seedling Morphology, Biomass Allocation and Physiology among Ten Temperate Tree Species in Response to Shade Is Related to Shade Tolerance and Not Leaf Habit’. *Plant Biology* 19, no. 2 (2017): 172–82. <https://doi.org/10.1111/plb.12531>.

Chong, C., W. Edwards, and M. Waycott. ‘Differences in Resprouting Ability Are Not Related to Seed Size or Seedling Growth in Four Riparian Woody Species’. *Journal of Ecology* 95, no. 4 (2007): 840–50. <https://doi.org/10.1111/j.1365-2745.2007.01241.x>.

Chou, F.-S., W.-C. Lin, Y.-H. Chen, and J.-B. Tsa. ‘Seed Fate of Castanopsis Indica (Fagaceae) in a Subtropical Evergreen Broadleaved Forest’. *Botanical Studies* 52, no. 3 (2011): 321–26.

Choung, Y., and M.S. Choung. ‘Biodiversity of Burned Forests Is Controlled by the Sprouting Ability of Prefire Species in Pinus Densiflora Forests’. *Ecological Engineering* 127 (2019): 356–62. <https://doi.org/10.1016/j.ecoleng.2018.12.016>.

Ciordia, M., M. De Lucas, V. Mateos, L. Rodríguez, J.C. García, and J. Majada. ‘Optimization of Germination Requirement and Seed Production of Wild-Type Vaccinium Myrtillus’, 715:85–90, 2006. <https://doi.org/10.17660/ActaHortic.2006.715.10>.

Clark, P.W., and A.W. D’Amato. ‘Seedbed Not Rescue Effect Buffer the Role of Extreme Precipitation on Temperate Forest Regeneration’. *Ecology* 104, no. 3 (2023). <https://doi.org/10.1002/ecy.3926>.

Clark, S.L., and S.E. Schlarbaum. ‘Effects of Acorn Size and Mass on Seedling Quality of Northern Red Oak (Quercus Rubra)’. *New Forests* 49, no. 4 (2018): 571–83. <https://doi.org/10.1007/s11056-018-9641-9>.

Colón, S.M., A.E. Lugo, and O.M. Ramos González. ‘Novel Dry Forests in Southwestern Puerto Rico’. *Forest Ecology and Management* 262, no. 2 (2011): 170–77. <https://doi.org/10.1016/j.foreco.2011.03.020>.

Connolly, B.M., L.K. Agnew, and J.L. Orrock. ‘Interactive Effects of Contact Fungicide and Cold Stratification on the Germination Rate for Five Dominant Temperate Tree Species’. *Forest Science* 63, no. 3 (2017): 303–9. <https://doi.org/10.5849/FS-2016-110R3>.

Cornelissen, J.H.C. ‘Interactive Effects of Season and Light Environment on Growth and Leaf Dynamics of Evergreen Tree Seedlings in the Humid Subtropics’. *Canadian Journal of Botany* 74, no. 4 (1996): 589–98. <https://doi.org/10.1139/b96-075>.

Cui, W.Y., S.J. Liu, Y.W. Wei, Y. Yin, L. Zhou, W.M. Zhou, and D.P. Yu. ‘Effects of Nitrogen Addition on Biomass Allocation of Pinus Koraiensis and Fraxinus Mandshurica Seedlings under Water Stress’. *Ying Yong Sheng Tai Xue Bao = The Journal of Applied Ecology* 30, no. 5 (2019): 1463–1453. <https://doi.org/10.13287/j.1001-9332.201905.027>.

Cullen, L.E., G.H. Stewart, R.P. Duncan, and J.G. Palmer. ‘Disturbance and Climate Warming Infiuences on New Zealand Nothofagus Tree-Line Population Dynamics’. *Journal of Ecology* 89, no. 6 (2001): 1061–71. <https://doi.org/10.1046/j.0022-0477>.

Da Silveira Bueno, R., E. Badalamenti, E. Barone, A. Cairone, A. La Mantia, G. Sala, and T. La Mantia. ‘First Assessment of Natural Regeneration and Seed Dispersal of Persian Walnut (Juglans Regia L.) in Mediterranean Agroecosystems’. *Arboriculture and Urban Forestry* 46, no. 3 (2020): 174–84.

Davis, M., and S. Smaill. ‘Mycorrhizal Colonisation of Exotic Conifers in Kānuka and Mānuka Shrublands’. *New Zealand Journal of Ecology* 33, no. 2 (2009): 147–55.

De Castanho, C.T., and P.I. Prado. ‘Benefit of Shading by Nurse Plant Does Not Change along a Stress Gradient in a Coastal Dune’. *PLoS ONE* 9, no. 8 (2014). <https://doi.org/10.1371/journal.pone.0105082>.

De Lombaerde, E., L. Baeten, K. Verheyen, M.P. Perring, S. Ma, and D. Landuyt. ‘Understorey Removal Effects on Tree Regeneration in Temperate Forests: A Meta-Analysis’. *Journal of Applied Ecology* 58, no. 1 (2021): 9–20. <https://doi.org/10.1111/1365-2664.13792>.

Del Tredici, P. ‘The Phenology of Sexual Reproduction in Ginkgo Biloba: Ecological and Evolutionary Implications’. *Botanical Review* 73, no. 4 (2007): 267–78. [https://doi.org/10.1663/0006-8101(2007)73[267:TPOSRI]2.0.CO;2](https://doi.org/10.1663/0006-8101(2007)73%5B267:TPOSRI%5D2.0.CO;2).

Devaney, J.L., P.M. Whelan, and M.A.K. Jansen. ‘Conspecific Negative Density Dependence in a Long-Lived Conifer, Yew Taxus Baccata L.’ *European Journal of Forest Research* 137, no. 1 (2018): 69–78. <https://doi.org/10.1007/s10342-017-1091-y>.

Döweler, F., B.S. Case, H.L. Buckley, and M.K.-F. Bader. ‘High Light-Induced Photoinhibition Is Not Limiting Seedling Establishment at Abrupt Treeline Ecotones in New Zealand’. *Tree Physiology* 41, no. 11 (2021): 2034–45. <https://doi.org/10.1093/treephys/tpab061>.

Drescher, M. ‘Snow Cover Manipulations and Passive Warming Affect Post-Winter Seed Germination: A Case Study of Three Cold-Temperate Tree Species’. *Climate Research* 60, no. 3 (2014): 175–86. <https://doi.org/10.3354/cr01237>.

Drescher, M., and S.C. Thomas. ‘Snow Cover Manipulations Alter Survival of Early Life Stages of Cold-Temperate Tree Species’. *Oikos* 122, no. 4 (2013): 541–54. <https://doi.org/10.1111/j.1600-0706.2012.20642.x>.

Drouin, M., R. Bradley, L. Lapointe, and J. Whalen. ‘Non-Native Anecic Earthworms (Lumbricus Terrestris L.) Reduce Seed Germination and Seedling Survival of Temperate and Boreal Trees Species’. *Applied Soil Ecology* 75 (2014): 145–49. <https://doi.org/10.1016/j.apsoil.2013.11.006>.

Dumais, D., and M. Prévost. ‘Management for Red Spruce Conservation in Québec: The Importance of Some Physiological and Ecological Characteristics - A Review’. *Forestry Chronicle* 83, no. 3 (2007): 378–92. <https://doi.org/10.5558/tfc83378-3>.

Dumais, D., P. Raymond, and M. Prévost. ‘Eight-Year Ecophysiology and Growth Dynamics of Picea Rubens Seedlings Planted in Harvest Gaps of Partially Cut Stands’. *Forest Ecology and Management* 478 (2020). <https://doi.org/10.1016/j.foreco.2020.118514>.

Dunn, G.M., J.R. Huth, and M.J. Lewty. ‘Coating Nursery Containers with Copper Carbonate Improves Root Morphology of Five Native Australian Tree Species Used in Agroforestry Systems’. *Agroforestry Systems* 37, no. 2 (1997): 143–55. <https://doi.org/10.1023/A:1005863707277>.

Dyderski, M.K., and A.M. Jagodziński. ‘Seedling Survival of Prunus Serotina Ehrh., Quercus Rubra L. and Robinia Pseudoacacia L. in Temperate Forests of Western Poland’. *Forest Ecology and Management* 450 (2019). <https://doi.org/10.1016/j.foreco.2019.117498>.

Fajardo, A., and F.I. Piper. ‘An Experimental Approach to Explain the Southern Andes Elevational Treeline’. *American Journal of Botany* 101, no. 5 (2014): 788–95. <https://doi.org/10.3732/ajb.1400166>.

Fisichelli, N., A. Wright, K. Rice, A. Mau, C. Buschena, and P.B. Reich. ‘First-Year Seedlings and Climate Change: Species-Specific Responses of 15 North American Tree Species’. *Oikos* 123, no. 11 (2014): 1331–40. <https://doi.org/10.1111/oik.01349>.

Forbes, A.S., D.A. Norton, and F.E. Carswell. ‘Tree Fern Competition Reduces Indigenous Forest Tree Seedling Growth within Exotic Pinus Radiata Plantations’. *Forest Ecology and Management* 359 (2016): 1–10. <https://doi.org/10.1016/j.foreco.2015.09.036>.

Frankland, B. ‘Effect of Gibberellic Acid, Kinetin and Other Substances on Seed Dormancy’. *Nature* 192, no. 4803 (1961): 678–79. <https://doi.org/10.1038/192678a0>.

Fyllas, N.M., P.I. Politi, A. Galanidis, P.G. Dimitrakopoulos, and M. Arianoutsou. ‘Simulating Regeneration and Vegetation Dynamics in Mediterranean Coniferous Forests’. *Ecological Modelling* 221, no. 11 (2010): 1494–1504. <https://doi.org/10.1016/j.ecolmodel.2010.03.003>.

Galíndez, G., T. Ledesma, A. Álvarez, V. Pastrana-Ignes, T. Bertuzzi, L. Lindow-López, S. Sühring, and P. Ortega-Baes. ‘Intraspecific Variation in Seed Germination and Storage Behaviour of Cordia Tree Species of Subtropical Montane Forests of Argentina: Implications for Ex Situ Conservation’. *South African Journal of Botany* 123 (2019): 393–99. <https://doi.org/10.1016/j.sajb.2019.03.029>.

García, D., J.R. Obeso, and I. Martínez. ‘Spatial Concordance between Seed Rain and Seedling Establishment in Bird-Dispersed Trees: Does Scale Matter?’ *Journal of Ecology* 93, no. 4 (2005): 693–704. <https://doi.org/10.1111/j.1365-2745.2005.01004.x>.

Gardiner, R., L.P. Shoo, and J.M. Dwyer. ‘Look to Seedling Heights, Rather than Functional Traits, to Explain Survival during Extreme Heat Stress in the Early Stages of Subtropical Rainforest Restoration’. *Journal of Applied Ecology* 56, no. 12 (2019): 2687–97. <https://doi.org/10.1111/1365-2664.13505>.

Gaxiola, A., L.E. Burrows, and D.A. Coomes. ‘Tree Fern Trunks Facilitate Seedling Regeneration in a Productive Lowland Temperate Rain Forest’. *Oecologia* 155, no. 2 (2008): 325–35. <https://doi.org/10.1007/s00442-007-0915-8>.

Gentle, C.B., and J.A. Duggin. ‘Allelopathy as a Competitive Strategy in Persistent Thickets of Lantana Camara L. in Three Australian Forest Communities’. *Plant Ecology* 132, no. 1 (1997): 85–95. <https://doi.org/10.1023/A:1009707404802>.

Gho-Illanes, D., C. Smith-Ramírez, I.A. Vásquez, and I. Díaz. ‘Frugivory of Persea Lingue (Lauraceae) and Its Effect on Seed Germination in Southern Chile’. *Gayana - Botanica* 72, no. 2 (2015): 250–57. <https://doi.org/10.4067/S0717-66432015000200009>.

Gómez-Aparicio, L., and C.D. Canham. ‘Neighbourhood Analyses of the Allelopathic Effects of the Invasive Tree Ailanthus Altissima in Temperate Forests’. *Journal of Ecology* 96, no. 3 (2008): 447–58. <https://doi.org/10.1111/j.1365-2745.2007.01352.x>.

Hadi, S.M.S., M.Z. Ahmed, A. Hameed, M.A. Khan, and B. Gul. ‘Seed Germination and Seedling Growth Responses of Toothbrush Tree (Salvadora Persica Linn.) to Different Interacting Abiotic Stresses’. *Flora: Morphology, Distribution, Functional Ecology of Plants* 243 (2018): 45–52. <https://doi.org/10.1016/j.flora.2018.04.002>.

Han, B., M.N. Umaña, X. Mi, X. Liu, L. Chen, Y. Wang, Y. Liang, W. Wei, and K. Ma. ‘The Role of Transcriptomes Linked with Responses to Light Environment on Seedling Mortality in a Subtropical Forest, China’. *Journal of Ecology* 105, no. 3 (2017): 592–601. <https://doi.org/10.1111/1365-2745.12760>.

Han, W., J. Jiang, Q. He, H. Huang, J. Hu, S. Hu, and J. Ni. ‘Effects of Nitrogen Deposition and Liming on the Early Regeneration of Two Dominant Tree Species in a Subtropical Forest of China’. *Ecoscience* 26, no. 3 (2019): 269–77. <https://doi.org/10.1080/11956860.2019.1570714>.

Hara, T., N. Nishimura, and S. Yamamoto. ‘Tree Competition and Species Coexistence in a Cool‐temperate Old‐growth Forest in Southwestern Japan’. *Journal of Vegetation Science* 6, no. 4 (1995): 565–74. <https://doi.org/10.2307/3236355>.

Harsch, M.A., R. Buxton, R.P. Duncan, P.E. Hulme, P. Wardle, and J. Wilmshurst. ‘Causes of Tree Line Stability: Stem Growth, Recruitment and Mortality Rates over 15 Years at New Zealand Nothofagus Tree Lines’. *Journal of Biogeography* 39, no. 11 (2012): 2061–71. <https://doi.org/10.1111/j.1365-2699.2012.02763.x>.

Hättenschwiler, S., and C. Körner. ‘Does Elevated CO2 Facilitate Naturalization of the Non-Indigenous Prunus Laurocerasus in Swiss Temperate Forests?’ *Functional Ecology* 17, no. 6 (2003): 778–85. <https://doi.org/10.1111/j.1365-2435.2003.00785.x>.

Hättenschwiler, S., and C. Korner. ‘Tree Seedling Responses to in Situ CO2-Enrichment Differ among Species and Depend on Understorey Light Availability’. *Global Change Biology* 6, no. 2 (2000): 213–26. <https://doi.org/10.1046/j.1365-2486.2000.00301.x>.

Hayamizu, M., Y. Nakata, and H. Torita. ‘Survival of Young, Dense Betula Ermanii Stands after Wildfire at Top Soil Removal Sites’. *Forestry* 95, no. 1 (2022): 130–41. <https://doi.org/10.1093/forestry/cpab024>.

Henry, C.R., and M.B. Walters. ‘Tree Species Size Class Patterns Portend Compositional Shifts and Low Resilience in Managed Northern Hardwood Forests’. *Ecosphere* 14, no. 7 (2023). <https://doi.org/10.1002/ecs2.4621>.

Hewitt, N. ‘Seed Size and Shade-Tolerance: A Comparative Analysis of North American Temperate Trees’. *Oecologia* 114, no. 3 (1998): 432–40. <https://doi.org/10.1007/s004420050467>.

Heyes, S.D., S.J. Sinclair, S.E. Hoebee, and J.W. Morgan. ‘How Widespread Are Recruitment Bottlenecks in Fragmented Populations of the Savanna Tree Banksia Marginata (Proteaceae)?’ *Plant Ecology* 221, no. 7 (2020): 545–57. <https://doi.org/10.1007/s11258-020-01033-0>.

Hille Ris Lambers, J., and J.S. Clark. ‘The Benefits of Seed Banking for Red Maple (Acer Rubrum): Maximizing Seedling Recruitment’. *Canadian Journal of Forest Research* 35, no. 4 (2005): 806–13. <https://doi.org/10.1139/x05-017>.

Hirayama, K., and M. Sakimoto. ‘Seedling Demography and Establishment of Cryptomeria Japonica in a Cool-Temperate, Old-Growth, Conifer Hardwood Forest in the Snowy Region of Japan’. *Journal of Forest Research* 10, no. 1 (2005): 67–71. <https://doi.org/10.1007/s10310-004-0104-0>.

Holík, J., and D. Janík. ‘Seed and Seedling Predation by Vertebrates Mediates the Effects of Adult Trees in Two Temperate Tree Species’. *Oecologia* 199, no. 3 (2022): 625–36. <https://doi.org/10.1007/s00442-022-05203-x>.

Holík, J., D. Janík, and D. Adam. ‘Light Can Modify Density-Dependent Seedling Mortality in a Temperate Forest’. *Journal of Vegetation Science* 32, no. 1 (2021). <https://doi.org/10.1111/jvs.12992>.

Hoshizaki, K., W. Suzuki, and T. Nakashizuka. ‘Evaluation of Secondary Dispersal in a Large-Seeded Tree Aesculus Turbinata: A Test of Directed Dispersal’. *Plant Ecology* 144, no. 2 (1999): 167–76. <https://doi.org/10.1023/A:1009816111057>.

Hosogi, D., and A. Kameyama. ‘Timing for the Planting Method Using Deciduous Forest Topsoil in Suburban Tokyo, Japan’. *Ecological Engineering* 26, no. 2 (2006): 123–31. <https://doi.org/10.1016/j.ecoleng.2005.06.014>.

Houle, G. ‘Seed Dispersal and Seedling Recruitment of Betula Alleghaniensis: Spatial Inconsistency in Time’. *Ecology* 79, no. 3 (1998): 807–18. <https://doi.org/10.2307/176580>.

Huang, L., C. Jin, L. Zhou, K. Song, S. Qian, D. Lin, L. Zhao, et al. ‘Benefit versus Cost Trade-Offs of Masting across Seed-to-Seedling Transition for a Dominant Subtropical Forest Species’. *Journal of Ecology* 109, no. 8 (2021): 3087–98. <https://doi.org/10.1111/1365-2745.13722>.

Hussain, A., I. Ahmed Qarshi, H. Nazir, I. Ullah, M. Rashid, and Z.K. Shinwari. ‘In Vitro Callogenesis and Organogenesis in Taxus Wallichiana Zucc. The Himalayan Yew’. *Pakistan Journal of Botany* 45, no. 5 (2013): 1755–59.

Ibell, P.T., Z. Xu, T. Blake, and T.J. Blumfield. ‘Effects of Weed Control and Fertilization at Early Establishment on Tree Nitrogen and Water Use in an Exotic F1 Hybrid Pine of Subtropical Australia’. *Journal of Soils and Sediments* 13, no. 9 (2013): 1538–52. <https://doi.org/10.1007/s11368-013-0765-4>.

Iqbal, K., A.K. Negi, N.A. Pala, and N.P. Todaria. ‘Seedling Recruitment of Rhododendron Arboreum: An Important NTFP Species of North-Western Himalaya, India’. *Ecological Questions* 34, no. 3 (2023). <https://doi.org/10.12775/EQ.2023.029>.

Ismael, A., J. Xue, D.F. Meason, J. Klápště, M. Gallart, Y. Li, P. Bellè, et al. ‘Genetic Variation in Drought-Tolerance Traits and Their Relationships to Growth in Pinus Radiata D. Don Under Water Stress’. *Frontiers in Plant Science* 12 (2022). <https://doi.org/10.3389/fpls.2021.766803>.

Itô, H., and T. Hino. ‘Effects of Deer and Mice on Seedling Survival in a Temperate Mixed Forest of Japan’. *Forest Ecology and Management* 256, no. 1–2 (2008): 129–35. <https://doi.org/10.1016/j.foreco.2008.04.016>.

Jang, W., C.R. Keyes, and J.-H. Lim. ‘Application of Mathematical Models in the Spatial Analysis of Early Tree Seedling Distribution Patterns within a Treefall Gap at Gwangneung Experimental Forest, Korea’. *Journal of Plant Biology* 56, no. 5 (2013): 283–89. <https://doi.org/10.1007/s12374-013-0044-3>.

Jin, M.R., Z. Wang, Z.S. He, L. Jiang, J.F. Liu, Y.Q. Lan, Y.W. Shi, and C.X. Shen. ‘Allelopathic Effect of Castanopsis Kawakamii Forest Litter on Seed Germination of Small Philippine Acacia (Acacia Confusa)’. *Applied Ecology and Environmental Research* 17, no. 6 (2019): 15103–16. <https://doi.org/10.15666/aeer/1706_1510315116>.

Kaye, M.W., and R.J. Wagner. ‘Eastern Deciduous Tree Seedlings Advance Spring Phenology in Response to Experimental Warming, but Not Wetting, Treatments’. *Plant Ecology* 215, no. 5 (2014): 543–54. <https://doi.org/10.1007/s11258-014-0322-2>.

Kelly, D., J.J. Ladley, and A.W. Robertson. ‘Is the Pollen-Limited Mistletoe Peraxilla Tetrapetala (Loranthaceae) Also Seed Limited?’ *Austral Ecology* 32, no. 8 (2007): 850–57. <https://doi.org/10.1111/j.1442-9993.2007.01765.x>.

Koga, W., T. Sasaki, K. Matsukura, K. Masaka, and K. Seiwa. ‘Roles of Pathogens and Mycorrhizae in Conspecific Negative Distance Dependency and Replacement of Tree Species in a Temperate Forest’. *Forest Ecology and Management* 544 (2023). <https://doi.org/10.1016/j.foreco.2023.121177>.

Koga, W., A. Suzuki, K. Masaka, and K. Seiwa. ‘Conspecific Distance-Dependent Seedling Performance, and Replacement of Conspecific Seedlings by Heterospecifics in Five Hardwood, Temperate Forest Species’. *Oecologia* 193, no. 4 (2020): 937–47. <https://doi.org/10.1007/s00442-020-04725-6>.

Kostel-Hughes, F., T.P. Young, and J.D. Wehr. ‘Effects of Leaf Litter Depth on the Emergence and Seedling Growth of Deciduous Forest Tree Species in Relation to Seed Size’. *Journal of the Torrey Botanical Society* 132, no. 1 (2005): 50–61. [https://doi.org/10.3159/1095-5674(2005)132[50:EOLLDO]2.0.CO;2](https://doi.org/10.3159/1095-5674(2005)132%5B50:EOLLDO%5D2.0.CO;2).

Kroschel, W.A., and S.L. King. ‘Floodplain Forest Tree Seedling Response to Variation in Flood Timing and Duration’. *Forest Ecology and Management* 502 (2021). <https://doi.org/10.1016/j.foreco.2021.119660>.

Kuang, X., K. Zhu, Z. Yuan, F. Lin, J. Ye, X. Wang, Y. Wang, and Z. Hao. ‘Conspecific Density Dependence and Community Structure: Insights from 11 Years of Monitoring in an Old-Growth Temperate Forest in Northeast China’. *Ecology and Evolution* 7, no. 14 (2017): 5191–5200. <https://doi.org/10.1002/ece3.3050>.

Kumar, M., P.-A. Waite, S.S. Paligi, and B. Schuldt. ‘Influence of Juvenile Growth on Xylem Safety and Efficiency in Three Temperate Tree Species’. *Forests* 13, no. 6 (2022). <https://doi.org/10.3390/f13060909>.

Kunstler, G., D.A. Coomes, and C.D. Canham. ‘Size-Dependence of Growth and Mortality Influence the Shade Tolerance of Trees in a Lowland Temperate Rain Forest’. *Journal of Ecology* 97, no. 4 (2009): 685–95. <https://doi.org/10.1111/j.1365-2745.2009.01482.x>.

Lacoretz, M.V., C. Malavert, N. Madanes, P. Cristiano, and P.M. Tognetti. ‘Seed Dormancy and Germination of Native and Invasive Alien Woody Species of an Endangered Temperate Forest in the Argentine Pampas’. *Forest Ecology and Management* 526 (2022). <https://doi.org/10.1016/j.foreco.2022.120577>.

Laughlin, D.C., and B.D. Clarkson. ‘Tree Seedling Survival Depends on Canopy Age, Cover and Initial Composition: Trade-Offs in Forest Restoration Enrichment Planting’. *Ecological Restoration* 36, no. 1 (2018): 52–61. <https://doi.org/10.3368/er.36.1.52>.

Lee, B.R., and I. Ibáñez. ‘Spring Phenological Escape Is Critical for the Survival of Temperate Tree Seedlings’. *Functional Ecology* 35, no. 8 (2021): 1848–61. <https://doi.org/10.1111/1365-2435.13821>.

Li, K., W. Dong, Y. Zhao, H. Xu, J. Chen, and C. Xu. ‘Effects of Cultivar and Ethanol Disinfection on Aseptic Germination of Loquat (Eriobotrya Japonica) Seeds’. *HortScience* 52, no. 7 (2017): 941–45. <https://doi.org/10.21273/HORTSCI11979-17>.

Li, Z., Z. Fu, S. Zhang, X. Zhang, X. Xue, Y. Chen, Z. Zhang, Z. Lai, and Y. Lin. ‘Genome-Wide Analysis of the GLP Gene Family and Overexpression of GLP1-5–1 to Promote Lignin Accumulation during Early Somatic Embryo Development in Dimocarpus Longan’. *BMC Genomics* 24, no. 1 (2023). <https://doi.org/10.1186/s12864-023-09201-y>.

Liang, H., L. Wang, Y. Wang, X. Quan, X. Li, Y. Xiao, and X. Yan. ‘Root Development in Cunninghamia Lanceolata and Schima Superba Seedlings Expresses Contrasting Preferences to Nitrogen Forms’. *Forests* 13, no. 12 (2022). <https://doi.org/10.3390/f13122085>.

Liang, M., X. Liu, R.S. Etienne, F. Huang, Y. Wang, and S. Yu. ‘Arbuscular Mycorrhizal Fungi Counteract the Janzen-Connell Effect of Soil Pathogens’. *Ecology* 96, no. 2 (2015): 562–74. <https://doi.org/10.1890/14-0871.1>.

Liang, M., L. Shi, D.F.R.P. Burslem, D. Johnson, M. Fang, X. Zhang, and S. Yu. ‘Soil Fungal Networks Moderate Density-Dependent Survival and Growth of Seedlings’. *New Phytologist* 230, no. 5 (2021): 2061–71. <https://doi.org/10.1111/nph.17237>.

Liang, X.Q., J. Liu, W.J. Ding, R.Y. Chang, and R.Q. Wang. ‘Effects of Simulated Acid Rain on Growth and Bleeding Sap Amount of Root in Quercus Mongolica’. *Shengtai Xuebao/ Acta Ecologica Sinica* 33, no. 15 (2013): 4583–90. <https://doi.org/10.5846/stxb201204270607>.

Liao, W., Z. Zhang, Z. Chen, C. Tang, and S. Deng. ‘Community Types, Phenology and Propagation Characteristics of Taxus Mairei in North Guangdong’. *Chinese Journal of Applied Ecology* 13, no. 7 (2002): 795–801.

Lin, F., L.S. Comita, X. Wang, X. Bai, Z. Yuan, D. Xing, and Z. Hao. ‘The Contribution of Understory Light Availability and Biotic Neighborhood to Seedling Survival in Secondary versus Old-Growth Temperate Forest’. *Plant Ecology* 215, no. 8 (2014): 795–807. <https://doi.org/10.1007/s11258-014-0332-0>.

Lin, W., J. Cai, and L. Xue. ‘Responses of Cinnamomum Camphora Seedling Growth and Leaf Traits to Additions of Nitrogen and Phosphorous under Different Planting Densities’. *Shengtai Xuebao* 39, no. 18 (2019): 6738–44. <https://doi.org/10.5846/stxb201811232545>.

Lines, E.R., M.A. Zavala, P. Ruiz-Benito, and D.A. Coomes. ‘Capturing Juvenile Tree Dynamics from Count Data Using Approximate Bayesian Computation’. *Ecography* 43, no. 3 (2020): 406–18. <https://doi.org/10.1111/ecog.04824>.

Liu, B., W. Chen, F.-S. Chen, R.-Y. Tang, X.-D. Wang, Y.-Q. Cheng, and W.-S. Bu. ‘Responses of Seedling Growth in Subtropical Secondary Broad-Leaved Forest to Nitrogen and Phosphorus Addition in Jiulian Mountain, China’. *Ying Yong Sheng Tai Xue Bao = The Journal of Applied Ecology* 31, no. 8 (2020): 2533–40. <https://doi.org/10.13287/j.1001-9332.202008.020>.

Liu, H., D.J. Johnson, Q. Yang, M. Xu, Z. Ma, X. Fang, Y. Shang, and X. Wang. ‘The Dynamics of Conspecific Tree and Seedling Neighbors on Seedling Survival in a Subtropical Forest’. *Forest Ecology and Management* 483 (2021). <https://doi.org/10.1016/j.foreco.2021.118924>.

Liu, H., G. Shen, Z. Ma, Q. Yang, J. Xia, X. Fang, and X. Wang. ‘Conspecific Leaf Litter-Mediated Effect of Conspecific Adult Neighborhood on Early-Stage Seedling Survival in A Subtropical Forest’. *Scientific Reports* 6 (2016). <https://doi.org/10.1038/srep37830>.

Liu, X., R.S. Etienne, M. Liang, Y. Wang, and S. Yu. ‘Experimental Evidence for an Intraspecific Janzen-Connell Effect Mediated by Soil Biota’. *Ecology* 96, no. 3 (2015): 662–71. <https://doi.org/10.1890/14-0014.1>.

Liu, X., M. Liang, R.S. Etienne, Y. Wang, C. Staehelin, and S. Yu. ‘Experimental Evidence for a Phylogenetic Janzen-Connell Effect in a Subtropical Forest’. *Ecology Letters* 15, no. 2 (2012): 111–18. <https://doi.org/10.1111/j.1461-0248.2011.01715.x>.

Liu, Y., S. Fang, P. Chesson, and F. He. ‘The Effect of Soil-Borne Pathogens Depends on the Abundance of Host Tree Species’. *Nature Communications* 6 (2015). <https://doi.org/10.1038/ncomms10017>.

Liu, Y., and F. He. ‘Warming Intensifies Soil Pathogen Negative Feedback on a Temperate Tree’. *New Phytologist* 231, no. 6 (2021): 2297–2307. <https://doi.org/10.1111/nph.17409>.

Liu, Y., S. Yu, Z.-P. Xie, and C. Staehelin. ‘Analysis of a Negative Plant-Soil Feedback in a Subtropical Monsoon Forest’. *Journal of Ecology* 100, no. 4 (2012): 1019–28. <https://doi.org/10.1111/j.1365-2745.2012.01953.x>.

Löf, M., J. Castro, M. Engman, A.B. Leverkus, P. Madsen, J.A. Reque, A. Villalobos, and E.S. Gardiner. ‘Tamm Review: Direct Seeding to Restore Oak (Quercus Spp.) Forests and Woodlands’. *Forest Ecology and Management* 448 (2019): 474–89. <https://doi.org/10.1016/j.foreco.2019.06.032>.

Lu, D., G.G. Wang, Q. Yan, T. Gao, and J. Zhu. ‘Effects of Gap Size and Within-Gap Position on Seedling Growth and Biomass Allocation: Is the Gap Partitioning Hypothesis Applicable to the Temperate Secondary Forest Ecosystems in Northeast China?’ *Forest Ecology and Management* 429 (2018): 351–62. <https://doi.org/10.1016/j.foreco.2018.07.031>.

Lu, J., D.J. Johnson, X. Qiao, Z. Lu, Q. Wang, and M. Jiang. ‘Density Dependence and Habitat Preference Shape Seedling Survival in a Subtropical Forest in Central China’. *Journal of Plant Ecology* 8, no. 6 (2014): 568–77. <https://doi.org/10.1093/jpe/rtv006>.

Lu, R., Y. Du, H. Sun, X. Xu, L. Yan, and J. Xia. ‘Nocturnal Warming Accelerates Drought-Induced Seedling Mortality of Two Evergreen Tree Species’. *Tree Physiology* 42, no. 6 (2022): 1164–76. <https://doi.org/10.1093/treephys/tpab168>.

Lu, Y., S. Ranjitkar, J.-C. Xu, X.-K. Ou, Y.-Z. Zhou, J.-F. Ye, X.-F. Wu, H. Weyerhaeuser, and J. He. ‘Propagation of Native Tree Species to Restore Subtropical Evergreen Broad-Leaved Forests in SW China’. *Forests* 7, no. 1 (2016). <https://doi.org/10.3390/f7010012>.

Luck, G.W. ‘The Demography and Cooperative Breeding Behaviour of the Rufous Treecreeper, Climacteris Rufa’. *Australian Journal of Zoology* 49, no. 5 (2001): 515–37. <https://doi.org/10.1071/ZO00087>.

Lusk, C.H., and A. Del Pozo. ‘Survival and Growth of Seedlings of 12 Chilean Rainforest Trees in Two Light Environments: Gas Exchange and Biomass Distribution Correlates’. *Austral Ecology* 27, no. 2 (2002): 173–82. <https://doi.org/10.1046/j.1442-9993.2002.01168.x>.

Lusk, C.H., T. Kaneko, E. Grierson, and M. Clearwater. ‘Correlates of Tree Species Sorting along a Temperature Gradient in New Zealand Rain Forests: Seedling Functional Traits, Growth and Shade Tolerance’. *Journal of Ecology* 101, no. 6 (2013): 1531–41. <https://doi.org/10.1111/1365-2745.12152>.

Lusk, C.H., K.M. Sendall, and P.J. Clarke. ‘Seedling Growth Rates and Light Requirements of Subtropical Rainforest Trees Associated with Basaltic and Rhyolitic Soils’. *Australian Journal of Botany* 62, no. 1 (2014): 48–55. <https://doi.org/10.1071/BT13262>.

Maguire, A.J., and R.K. Kobe. ‘Drought and Shade Deplete Nonstructural Carbohydrate Reserves in Seedlings of Five Temperate Tree Species’. *Ecology and Evolution* 5, no. 23 (2015): 5711–21. <https://doi.org/10.1002/ece3.1819>.

Marden, M., S. Lambie, and C. Phillips. ‘Potential Effectiveness of Low-Density Plantings of Mānuka (Leptospermum Scoparium) as an Erosion Mitigation Strategy in Steeplands, Northern Hawke’s Bay, New Zealand’. *New Zealand Journal of Forestry Science* 50 (2020): 1–25. <https://doi.org/10.33494/nzjfs502020x82x>.

Martin, P., A.C. Newton, E. Cantarello, and P.M. Evans. ‘Analysis of Ecological Thresholds in a Temperate Forest Undergoing Dieback’. *PLoS ONE* 12, no. 12 (2017). <https://doi.org/10.1371/journal.pone.0189578>.

Martini, F., C.-H. Chang-Yang, and I.-F. Sun. ‘Variation in Biotic Interactions Mediates the Effects of Masting and Rainfall Fluctuations on Seedling Demography in a Subtropical Rainforest’. *Journal of Ecology* 110, no. 4 (2022): 762–71. <https://doi.org/10.1111/1365-2745.13833>.

Masaki, T., K. Osumi, K. Takahashi, and K. Hozshizaki. ‘Seedling Dynamics of Acer Mono and Fagus Crenata: An Environmental Filter Limiting Their Adult Distributions’. *Plant Ecology* 177, no. 2 (2005): 189–99. <https://doi.org/10.1007/s11258-005-2177-z>.

Mathiasen, P., A.E. Rovere, and A.C. Premoli. ‘Genetic Structure and Early Effects of Inbreeding in Fragmented Temperate Forests of a Self-Incompatible Tree, Embothrium Coccineum’. *Conservation Biology* 21, no. 1 (2007): 232–40. <https://doi.org/10.1111/j.1523-1739.2006.00565.x>.

McAlpine, K.G., and D.R. Drake. ‘The Effects of Small-Scale Environmental Heterogeneity on Seed Germination in Experimental Treefall Gaps in New Zealand’. *Plant Ecology* 165, no. 2 (2003): 207–15. <https://doi.org/10.1023/A:1022247707932>.

Mccarthy-Neumann, S., and I. Ibáñez. ‘Tree Range Expansion May Be Enhanced by Escape from Negative Plant-Soil Feedbacks’. *Ecology* 93, no. 12 (2012): 2637–49. <https://doi.org/10.1890/11-2281.1>.

McCarthy-Neumann, S., and R.K. Kobe. ‘Conspecific and Heterospecific Plant-Soil Feedbacks Influence Survivorship and Growth of Temperate Tree Seedlings’. *Journal of Ecology* 98, no. 2 (2010): 408–18. <https://doi.org/10.1111/j.1365-2745.2009.01620.x>.

McConnaughay, K.D.M., S.L. Bassow, G.M. Berntson, and F.A. Bazzaz. ‘Leaf Senescence and Decline of End-of-Season Gas Exchange in Five Temperate Deciduous Tree Species Grown in Elevated CO2 Concentrations’. *Global Change Biology* 2, no. 1 (1996): 25–33. <https://doi.org/10.1111/j.1365-2486.1996.tb00046.x>.

Mcconnaughay, K.D.M., A.B. Nicotra, and F.A. Bazzaz. ‘Rooting Volume, Nutrient Availability, and CO2-Induced Growth Enhancements in Temperate Forest Tree Seedlings’. *Ecological Applications* 6, no. 2 (1996): 619–27. <https://doi.org/10.2307/2269396>.

Meeussen, C., K. De Pauw, P. Sanczuk, J. Brunet, S.A.O. Cousins, C. Gasperini, P.-O. Hedwall, et al. ‘Initial Oak Regeneration Responses to Experimental Warming along Microclimatic and Macroclimatic Gradients’. *Plant Biology* 24, no. 5 (2022): 745–57. <https://doi.org/10.1111/plb.13412>.

Meng, L., C. Zhang, J. Yao, and X. Zhao. ‘Effects of Density and Habitat on Arbor Seedling Survival in a Mixed Conifer and Broad-Leaved Forest in Jiaohe, Jilin Province’. *Linye Kexue/Scientia Silvae Sinicae* 55, no. 11 (2019): 172–80. <https://doi.org/10.11707/j.1001-7488.20191119>.

Michel, P., D.J. Burritt, and W.G. Lee. ‘Bryophytes Display Allelopathic Interactions with Tree Species in Native Forest Ecosystems’. *Oikos* 120, no. 8 (2011): 1272–80. <https://doi.org/10.1111/j.1600-0706.2010.19148.x>.

Mir, N.A., T.H. Masoodi, M. Asif, H.A. Bhat, A.-U. Nabi, A.A. Gatoo, and A.A. Parrey. ‘Seed Technology in High Altitude Conifers with Special Reference to Temperate Conifers of Kashmir Valley’. *Ecology, Environment and Conservation* 20, no. 3 (2014): 1089–99.

Mishima, K., H. Hirakawa, T. Iki, Y. Fukuda, T. Hirao, A. Tamura, and M. Takahashi. ‘Comprehensive Collection of Genes and Comparative Analysis of Full-Length Transcriptome Sequences from Japanese Larch (Larix Kaempferi) and Kuril Larch (Larix Gmelinii Var. Japonica)’. *BMC Plant Biology* 22, no. 1 (2022). <https://doi.org/10.1186/s12870-022-03862-9>.

Modrzyński, J., D.J. Chmura, M.G. Tjoelker, and S. Thomas. ‘Seedling Growth and Biomass Allocation in Relation to Leaf Habit and Shade Tolerance among 10 Temperate Tree Species’. *Tree Physiology* 35, no. 8 (2015): 879–93. <https://doi.org/10.1093/treephys/tpv053>.

Muffler, L., J. Schmeddes, R. Weigel, A. Barbeta, I. Beil, A. Bolte, C. Buhk, et al. ‘High Plasticity in Germination and Establishment Success in the Dominant Forest Tree Fagus Sylvatica across Europe’. *Global Ecology and Biogeography* 30, no. 8 (2021): 1583–96. <https://doi.org/10.1111/geb.13320>.

Mugwedi, L., M. Rouget, B. Egoh, S. Sershen, S. Ramdhani, R. Slotow, and H.P. Moyo. ‘Can an El Niño Induced Drought Hamper the Reforestation of the Subtropical Forest?’ *South African Journal of Botany* 141 (2021): 152–57. <https://doi.org/10.1016/j.sajb.2021.04.024>.

Muscolo, A., G. Settineri, S. Bagnato, R. Mercurio, and M. Sidari. ‘Use of Canopy Gap Openings to Restore Coniferous Stands in Mediterranean Environment’. *IForest* 10, no. 1 (2017): 322–27. <https://doi.org/10.3832/ifor1983-009>.

Nadolny, C. ‘Recruitment and Survival of Argyrodendron Actinophyllum Seedlings in an Australian Rainforest’. *Austral Ecology* 24, no. 3 (1999): 258–69. <https://doi.org/10.1046/j.1442-9993.1999.00970.x>.

Niu, J., W. Zhang, Z. Feng, X. Wang, and Y. Tian. ‘Impact of Elevated O 3 on Visible Foliar Symptom, Growth and Biomass of Cinnamomum Camphora Seedlings under Different Nitrogen Loads’. *Journal of Environmental Monitoring* 13, no. 10 (2011): 2873–79. <https://doi.org/10.1039/c1em10305a>.

Obeso, J.R., I. Martínez, and D. García. ‘Seed Size Is Heterogeneously Distributed among Destination Habitats in Animal Dispersed Plants’. *Basic and Applied Ecology* 12, no. 2 (2011): 134–40. <https://doi.org/10.1016/j.baae.2011.01.003>.

O’Hanlon, R. ‘Below-Ground Ectomycorrhizal Communities: The Effect of Small Scale Spatial and Short Term Temporal Variation’. *Symbiosis* 57, no. 2 (2012): 57–71. <https://doi.org/10.1007/s13199-012-0179-x>.

Osugi, S., B.E. Trentin, and S. Koike. ‘What Determines the Seedling Viability of Different Tree Species in Raccoon Dog Latrines?’ *Acta Oecologica* 106 (2020). <https://doi.org/10.1016/j.actao.2020.103604>.

Osunkoya, O.O., and R.G. Creese. ‘Population Structure, Spatial Pattern and Seedling Establishment of the Grey Mangrove, Avicennia Marina Var. Australasica, in New Zealand’. *Australian Journal of Botany* 45, no. 4 (1997): 707–25. <https://doi.org/10.1071/BT96070>.

Pan, Y., Z. Yao, N. Zhang, and G.F. Veen. ‘The Role of Soil-Borne Fungi in Driving the Coexistence of Pinus Massoniana and Lithocarpus Glaber in a Subtropical Forest via Plant-Soil Feedback’. *Journal of Plant Ecology* 14, no. 6 (2021): 1189–1203. <https://doi.org/10.1093/jpe/rtab058>.

Papaik, M.J., and C.D. Canham. ‘Species Resistance and Community Response to Wind Disturbance Regimes in Northern Temperate Forests’. *Journal of Ecology* 94, no. 5 (2006): 1011–26. <https://doi.org/10.1111/j.1365-2745.2006.01153.x>.

Parsons, R.F. ‘Adaptations to Floodplains in Populus and Salix: The Role of Collet Hairs’. *Trees - Structure and Function* 27, no. 1 (2013): 1–5. <https://doi.org/10.1007/s00468-012-0738-z>.

Paul, M., C.P. Catterall, P.C. Pollard, and J. Kanowski. ‘Does Soil Variation between Rainforest, Pasture and Different Reforestation Pathways Affect the Early Growth of Rainforest Pioneer Species?’ *Forest Ecology and Management* 260, no. 3 (2010): 370–77. <https://doi.org/10.1016/j.foreco.2010.04.029>.

Paul, S., S.S. Samant, M. Lal, and J. Ram. ‘Population Assessment and Ecological Niche Modelling of Carpinus Viminea Wall. Ex Lindl. - A Multipurpose Tree for Conservation in the Indian Himalayan Region’, 84:681–94, 2018. <https://www.scopus.com/inward/record.uri?eid=2-s2.0-85053236369&partnerID=40&md5=82ed95ec2399684cf7173025ea8aca7d>.

Pawłowski, T.A. ‘Proteomic Approach to Analyze Dormancy Breaking of Tree Seeds’. *Plant Molecular Biology* 73, no. 1–2 (2010): 15–25. <https://doi.org/10.1007/s11103-010-9623-6>.

Pegman, A.P.M., G.L.W. Perry, and M.N. Clout. ‘Size-Based Fruit Selection by a Keystone Avian Frugivore and Effects on Seed Viability’. *New Zealand Journal of Botany* 55, no. 2 (2017): 118–33. <https://doi.org/10.1080/0028825X.2016.1247882>.

Percy, K. ‘The effects of simulated acid rain on germinative capacity, growth and morphology of forest tree seedlings’. *New Phytologist* 104, no. 3 (1986): 473–84. <https://doi.org/10.1111/j.1469-8137.1986.tb02914.x>.

Pérez-Ruiz, C.L., E.I. Badano, J.P. Rodas-Ortiz, P. Delgado-Sánchez, J. Flores, D. Douterlungne, and J.A. Flores-Cano. ‘Climate Change in Forest Ecosystems: A Field Experiment Addressing the Effects of Raising Temperature and Reduced Rainfall on Early Life Cycle Stages of Oaks’. *Acta Oecologica* 92 (2018): 35–43. <https://doi.org/10.1016/j.actao.2018.08.006>.

Pio, R., E.A. Chagas, W. Barbosa, A.F.C. Tombolato, and G. Signorini. ‘Intergeneric Grafting of Pear Cultivars to the “Japonês” Quince Tree’, 800 PART 2:707–11, 2008. <https://doi.org/10.17660/actahortic.2008.800.96>.

Plotkin, A.B., D. Foster, J. Carlson, and A. Magill. ‘Survivors, Not Invaders, Control Forest Development Following Simulated Hurricane’. *Ecology* 94, no. 2 (2013): 414–23. <https://doi.org/10.1890/12-0487.1>.

Portsmuth, A., and U. Niinemets. ‘Interacting Controls by Light Availability and Nutrient Supply on Biomass Allocation and Growth of Betula Pendula and B. Pubescens Seedlings’. *Forest Ecology and Management* 227, no. 1–2 (2006): 122–34. <https://doi.org/10.1016/j.foreco.2006.02.020>.

Qin, J., X. Yue, X. Shang, and S. Fang. ‘Nitrogen Forms Alter Triterpenoid Accumulation and Related Gene Expression in Cyclocarya Paliurus (Batalin) Iljinsk. Seedlings’. *Forests* 11, no. 6 (2020). <https://doi.org/10.3390/F11060631>.

Qu, L., H.J. De Boeck, H. Fan, G. Dong, J. Chen, W. Xu, Z. Ge, Z. Huang, C. Shao, and Y. Hu. ‘Diverging Responses of Two Subtropical Tree Species (Schima Superba and Cunninghamia Lanceolata) to Heat Waves’. *Forests* 11, no. 5 (2020). <https://doi.org/10.3390/F11050513>.

Ramsey, D.S.L., D.M. Forsyth, C.J. Veltman, S.J. Nicol, C.R. Todd, R.B. Allen, W.J. Allen, et al. ‘An Approximate Bayesian Algorithm for Training Fuzzy Cognitive Map Models of Forest Responses to Deer Control in a New Zealand Adaptive Management Experiment’. *Ecological Modelling* 240 (2012): 93–104. <https://doi.org/10.1016/j.ecolmodel.2012.04.022>.

Rao, P., S.K. Barik, H.N. Pandey, and R.S. Tripathi. ‘Tree Seed Germination and Seedling Establishment in Treefall Gaps and Understorey in a Subtropical Forest of Northeast India’. *Austral Ecology* 22, no. 2 (1997): 136–45. <https://doi.org/10.1111/j.1442-9993.1997.tb00652.x>.

Royo, A.A., and W.P. Carson. ‘Stasis in Forest Regeneration Following Deer Exclusion and Understory Gap Creation: A 10-Year Experiment’. *Ecological Applications* 32, no. 4 (2022). <https://doi.org/10.1002/eap.2569>.

Rozendaal, D.M.A., and R.K. Kobe. ‘A Forest Tent Caterpillar Outbreak Increased Resource Levels and Seedling Growth in a Northern Hardwood Forest’. *PLoS ONE* 11, no. 11 (2016). <https://doi.org/10.1371/journal.pone.0167139>.

Ruscoe, W.A., J.S. Elkinton, D. Choquenot, and R.B. Allen. ‘Predation of Beech Seed by Mice: Effects of Numerical and Functional Responses’. *Journal of Animal Ecology* 74, no. 6 (2005): 1005–19. <https://doi.org/10.1111/j.1365-2656.2005.00998.x>.

Salinas, F., and J.J. Armesto. ‘Regeneration Niche of Three Epiphytic Species of Gesneriaceae from Chilean Rainforests: Implications for the Evolution of Growth Habits in Coronanthereae’. *Botanical Journal of the Linnean Society* 170, no. 1 (2012): 79–92. <https://doi.org/10.1111/j.1095-8339.2012.01256.x>.

Sánchez-Gómez, D., M.A. Zavala, and F. Valladares. ‘Seedling Survival Responses to Irradiance Are Differentially Influenced by Low-Water Availability in Four Tree Species of the Iberian Cool Temperate-Mediterranean Ecotone’. *Acta Oecologica* 30, no. 3 (2006): 322–32. <https://doi.org/10.1016/j.actao.2006.05.005>.

Sapkota, I.P., and P.C. Odén. ‘Gap Characteristics and Their Effects on Regeneration, Dominance and Early Growth of Woody Species’. *Journal of Plant Ecology* 2, no. 1 (2009): 21–29. <https://doi.org/10.1093/jpe/rtp004>.

Sayyad-Amin, P., and A.-R. Shahsavar. ‘Improvement of Seed Germination of Date-Plum (Diospyros Lotus l.) by Physical and Chemical Treatments’. *Journal of Chemical Health Risks* 9, no. 1 (2019): 51–56. <https://doi.org/10.22034/jchr.2019.664164>.

Schlindwein, G., C.C.D. Schlindwein, and L.R. Dillenburg. ‘Seasonal Cycle of Seed Dormancy Controls the Recruitment of Butia Odorata (ARECACEAE) Seedlings in Savanna-like Palm Tree Formations in Southern Brazil’. *Austral Ecology* 44, no. 8 (2019): 1398–1409. <https://doi.org/10.1111/aec.12813>.

Schmidt, S., L.L. Handley, and T. Sangtiean. ‘Effects of Nitrogen Source and Ectomycorrhizal Association on Growth and δ15N of Two Subtropical Eucalyptus Species from Contrasting Ecosystems’. *Functional Plant Biology* 33, no. 4 (2006): 367–79. <https://doi.org/10.1071/FP05260>.

Schnurr, J.L., C.D. Canham, R.S. Ostfeld, and R.S. Inouye. ‘Neighborhood Analyses of Small-Mammal Dynamics: Impacts on Seed Predation and Seedling Establishment’. *Ecology* 85, no. 3 (2004): 741–55. <https://doi.org/10.1890/02-0644>.

Seifert, J.R., D.F. Jacobs, and M.F. Selig. ‘Influence of Seasonal Planting Date on Field Performance of Six Temperate Deciduous Forest Tree Species’. *Forest Ecology and Management* 223, no. 1–3 (2006): 371–78. <https://doi.org/10.1016/j.foreco.2005.11.019>.

Seiwa, K. ‘Trade-Offs between Seedling Growth and Survival in Deciduous Broadleaved Trees in a Temperate Forest’. *Annals of Botany* 99, no. 3 (2007): 537–44. <https://doi.org/10.1093/aob/mcl283>.

Sendall, K.M., C.H. Lusk, and P.B. Reich. ‘Trade-Offs in Juvenile Growth Potential vs. Shade Tolerance among Subtropical Rain Forest Trees on Soils of Contrasting Fertility’. *Functional Ecology* 30, no. 6 (2016): 845–55. <https://doi.org/10.1111/1365-2435.12573>.

Sendall, K.M., P.B. Reich, and C.H. Lusk. ‘Size-Related Shifts in Carbon Gain and Growth Responses to Light Differ among Rainforest Evergreens of Contrasting Shade Tolerance’. *Oecologia* 187, no. 3 (2018): 609–23. <https://doi.org/10.1007/s00442-018-4125-3>.

Shang, H., Y. Wang, B. Han, F.J. Bongers, X. Mi, L. Chen, Y. Liang, and K. Ma. ‘Molecular Defense Responses to Natural Enemies Determine Seedling Survival in a Subtropical Forest’. *Forest Ecology and Management* 544 (2023). <https://doi.org/10.1016/j.foreco.2023.121191>.

Shang, H., Y. Wang, B. Han, X. Mi, L. Chen, Y. Liang, and K. Ma. ‘Effects of Functional Phylogeny of Light-Response-Related Orthologous Genes on Seedling Survival in a Subtropical Forest’. *Forest Ecosystems* 10 (2023). <https://doi.org/10.1016/j.fecs.2023.100087>.

Shen, Z., S. Guo, Y. Yang, and X. Yi. ‘Decrease of Large-Bodied Dispersers Limits Recruitment of Large-Seeded Trees but Benefits Small-Seeded Trees’. *Israel Journal of Ecology and Evolution* 58, no. 1 (2012): 53–67. <https://doi.org/10.1560/IJEE.58.1.53>.

Shimoda, K., K. Kimura, M. Kanzaki, and K. Yoda. ‘The Regeneration of Pioneer Tree Species under Browsing Pressure of Sika Deer in an Evergreen Oak Forest’. *Ecological Research* 9, no. 1 (1994): 85–92. <https://doi.org/10.1007/BF02347245>.

Singh, O., and V. Rattan. ‘Allelopathic Effects of Viburnum Nervosum on Seed Germination and Seedling Growth of Abies Pindrow Spach’. *Allelopathy Journal* 32, no. 1 (2013): 113–22.

Sommerville, K.E., and J. Read. ‘Contrasting Water-Use Strategies in Two Sympatric Cool-Temperate Rainforest Species, Nothofagus Cunninghamii (Nothofagaceae) and Atherosperma Moschatum (Atherospermataceae)’. *Australian Journal of Botany* 56, no. 2 (2008): 109–18. <https://doi.org/10.1071/BT07138>.

Song, X., J. Yang, M. Cao, L. Lin, Z. Sun, H. Wen, and N.G. Swenson. ‘Traits Mediate a Trade-off in Seedling Growth Response to Light and Conspecific Density in a Diverse Subtropical Forest’. *Journal of Ecology* 109, no. 2 (2021): 703–13. <https://doi.org/10.1111/1365-2745.13497>.

Song, Y.-G., M. Deng, and Y.-T. Wang. ‘Germination Characteristics of Quercus Virginiana Seeds’. *Chinese Journal of Ecology* 34, no. 5 (2015): 1295–1300.

Standish, R.J., A.W. Robertson, and P.A. Williams. ‘The Impact of an Invasive Weed Tradescantia Fluminensis on Native Forest Regeneration’. *Journal of Applied Ecology* 38, no. 6 (2001): 1253–63. <https://doi.org/10.1046/j.0021-8901.2001.00673.x>.

Stokes, D.J., T.R. Healy, and P.J. Cooke. ‘Expansion Dynamics of Monospecific, Temperate Mangroves and Sedimentation in Two Embayments of a Barrier-Enclosed Lagoon, Tauranga Harbour, New Zealand’. *Journal of Coastal Research* 26, no. 1 (2010): 113–22. <https://doi.org/10.2112/08-1043.1>.

Stone, D.E., S.-H. Oh, E.A. Tripp, L.E. Ros G, and P.S. Manos. ‘Natural History, Distribution, Phylogenetic Relationships, and Conservation of Central American Black Walnuts (Juglans Sect. Rhysocaryon)’. *Journal of the Torrey Botanical Society* 136, no. 1 (2009): 1–25. <https://doi.org/10.3159/08-RA-036R.1>.

Stromberg, J.C. ‘Flood Flows and Population Dynamics of Arizona Sycamore (Platanus Wrightii)’. *Western North American Naturalist* 62, no. 2 (2002): 170–87.

Suarez, M.L., and Y. Sasal. ‘Drought-Induced Mortality Affects Understory Vegetation: Release after Death’. *Ecological Research* 27, no. 4 (2012): 715–24. <https://doi.org/10.1007/s11284-012-0945-5>.

Sujeeun, L., and S.C. Thomas. ‘Biochar Mitigates Allelopathic Effects in Temperate Trees’. *Ecological Applications* 33, no. 4 (2023). <https://doi.org/10.1002/eap.2832>.

Suzuki, K. ‘Environmental Conditions after Seed Dispersal of Spring Ephemeral Species at the Floor of Deciduous, Broad-Leaved Forests’. In *From Seed Germination to Young Plants: Ecology, Growth and Environmental Influences*, 329–40, 2013. <https://www.scopus.com/inward/record.uri?eid=2-s2.0-84895396732&partnerID=40&md5=00b489cb9df96a7725361f0683316657>.

Tang, H., Y.-Y. Hu, W.-W. Yu, L.-L. Song, and J.-S. Wu. ‘Growth, Photosynthetic and Physiological Responses of Torreya Grandis Seedlings to Varied Light Environments’. *Trees - Structure and Function* 29, no. 4 (2015): 1011–22. <https://doi.org/10.1007/s00468-015-1180-9>.

Tang, Y., K. Zhang, Y. Zhang, and J. Tao. ‘Dormancy-Breaking and Germination Requirements for Seeds of Sorbus Alnifolia (Siebold & Zucc.) K.Koch (Rosaceae), a Mesic Forest Tree with High Ornamental Potential’. *Forests* 10, no. 4 (2019). <https://doi.org/10.3390/f10040319>.

Thomas, S.C. ‘Biochar Effects on Germination and Radicle Extension in Temperate Tree Seedlings under Field Conditions’. *Canadian Journal of Forest Research* 51, no. 1 (2021): 10–17. <https://doi.org/10.1139/cjfr-2019-0386>.

Tonn, N., and I. Ibáñez. ‘Plant-Mycorrhizal Fungi Associations along an Urbanization Gradient: Implications for Tree Seedling Survival’. *Urban Ecosystems* 20, no. 4 (2017): 823–37. <https://doi.org/10.1007/s11252-016-0630-5>.

Tseng, M.-H., W.-R. Lai, C.-L. Hsieh, and Y.-H. Kuo. ‘Allelopathy on Bark of Downed Logs of Chamaecyparis Obtusa Sieb. and Zucc. Var. Formosana (Hayata) Rehder’. *Journal of Chemical Ecology* 33, no. 6 (2007): 1283–96. <https://doi.org/10.1007/s10886-007-9278-1>.

Umaña, M.N., G. Arellano, N.G. Swenson, and J. Zambrano. ‘Tree Seedling Trait Optimization and Growth in Response to Local-Scale Soil and Light Variability’. *Ecology* 102, no. 4 (2021). <https://doi.org/10.1002/ecy.3252>.

Uno, H., Y. Inatomi, M. Ueno, and H. Iijima. ‘Effects of Sika Deer (Cervus Nippon) and Dwarf Bamboo (Sasa Senanensis) on Tree Seedlings in a Cool-Temperate Mixed Forest on Hokkaido Island, Japan’. *European Journal of Forest Research* 138, no. 6 (2019): 929–38. <https://doi.org/10.1007/s10342-019-01214-1>.

Van Roon, M.R., and T.P.M. Rigold. ‘Sustainability and Coincidence of Riparian Vegetation and In-Stream Macroinvertebrate Communities in Auckland’s Water Sensitive Developments’, 2012. <https://www.scopus.com/inward/record.uri?eid=2-s2.0-85099492148&partnerID=40&md5=82adf60cea0c44b71bde35ec9ffccde0>.

Velazco, S.J.E., C.T. Blum, and P.M. Hoffmann. ‘Germination and Seedlings Development of the Threatened Species Quillaja Brasiliensis’. *Cerne* 24, no. 2 (2018): 90–97. <https://doi.org/10.1590/01047760201824022530>.

Viswanath, S., R.P. Singh, and R.C. Thapliyal. ‘Seed Germination Patterns in a Himalayan Moist Temperate Forest’. *Tropical Ecology* 43, no. 2 (2002): 265–73.

Von Arx, G., E. Graf Pannatier, A. Thimonier, and M. Rebetez. ‘Microclimate in Forests with Varying Leaf Area Index and Soil Moisture: Potential Implications for Seedling Establishment in a Changing Climate’. *Journal of Ecology* 101, no. 5 (2013): 1201–13. <https://doi.org/10.1111/1365-2745.12121>.

Walker, L.R., B.D. Clarkson, W.B. Silvester, and B.R. Clarkson. ‘Colonization Dynamics and Facilitative Impacts of a Nitrogen-Fixing Shrub in Primary Succession’. *Journal of Vegetation Science* 14, no. 2 (2003): 277–90. <https://doi.org/10.1111/j.1654-1103.2003.tb02153.x>.

Walters, M.B., E.J. Farinosi, and J.L. Willis. ‘Deer Browsing and Shrub Competition Set Sapling Recruitment Height and Interact with Light to Shape Recruitment Niches for Temperate Forest Tree Species’. *Forest Ecology and Management* 467 (2020). <https://doi.org/10.1016/j.foreco.2020.118134>.

Walters, M.B., J.M. Kunkle, R.K. Kobe, and E.J. Farinosi. ‘Seedling Drought Responses Governed by Root Traits, Site-Soil Moisture Regimes and Overstory Competition-Facilitation’. *Forest Ecology and Management* 544 (2023). <https://doi.org/10.1016/j.foreco.2023.121159>.

Wan, D., J.-M. Liu, Y.-Z. Xu, M.-X. Jiang, Z.-R. Gu, and C.-L. Liao. ‘Study on Variations of Seedling Functional Traits in A Mixed Evergreen and Deciduous Broad-Leaved Forest in Central China’. *Resources and Environment in the Yangtze Basin* 29, no. 4 (2020): 938–49. <https://doi.org/10.11870/cjlyzyyhj202004015>.

Wandrag, E.M., A. Sheppard, R.P. Duncan, and P.E. Hulme. ‘Reduced Availability of Rhizobia Limits the Performance but Not Invasiveness of Introduced Acacia’. *Journal of Ecology* 101, no. 5 (2013): 1103–13. <https://doi.org/10.1111/1365-2745.12126>.

Wang, B.S.P., and P. Berjak. ‘Beneficial Effects of Moist Chilling on the Seeds of Black Spruce (Picea Mariana [Mill.] B.S.P.)’. *Annals of Botany* 86, no. 1 (2000): 29–36. <https://doi.org/10.1006/anbo.2000.1150>.

Wang, J., D. Hui, H. Ren, N. Liu, Z. Sun, L. Yang, and H. Lu. ‘Short-Term Canopy and Understory Nitrogen Addition Differ in Their Effects on Seedlings of Dominant Woody Species in a Subtropical Evergreen Broadleaved Forest’. *Global Ecology and Conservation* 31 (2021). <https://doi.org/10.1016/j.gecco.2021.e01855>.

Wang, M., W.-W. Zhang, N. Li, Y.-Y. Liu, X.-B. Zheng, and G.-Y. Hao. ‘Photosynthesis and Growth Responses of Fraxinus Mandshurica Rupr. Seedlings to a Gradient of Simulated Nitrogen Deposition’. *Annals of Forest Science* 75, no. 1 (2018). <https://doi.org/10.1007/s13595-017-0678-2>.

Wang, R., X. Zhang, Y.-S. Shi, Y.-Y. Li, J. Wu, F. He, and X.-Y. Chen. ‘Habitat Fragmentation Changes Top-down and Bottom-up Controls of Food Webs’. *Ecology* 101, no. 8 (2020). <https://doi.org/10.1002/ecy.3062>.

Wang, Y., M.W. Cadotte, J. Chen, X. Mi, H. Ren, X. Liu, M. Yu, J. Zhang, and K. Ma. ‘Neighborhood Interactions on Seedling Survival Were Greatly Altered Following an Extreme Winter Storm’. *Forest Ecology and Management* 461 (2020). <https://doi.org/10.1016/j.foreco.2020.117940>.

Washitani, I., and Y. Tang. ‘Microsite Variation in Light Availability and Seedling Growth of Quercus Serrata in a Temperate Pine Forest’. *Ecological Research* 6, no. 3 (1991): 305–16. <https://doi.org/10.1007/BF02347130>.

Wayne, P.M., E.G. Reekie, and F.A. Bazzaz. ‘Elevated CO2 Ameliorates Birch Response to High Temperature and Frost Stress: Implications for Modeling Climate-Induced Geographic Range Shifts’. *Oecologia* 114, no. 3 (1998): 335–42. <https://doi.org/10.1007/s004420050455>.

Webster, C.R., K. Nelson, and S.R. Wangen. ‘Stand Dynamics of an Insular Population of an Invasive Tree, Acer Platanoides’. *Forest Ecology and Management* 208, no. 1–3 (2005): 85–99. <https://doi.org/10.1016/j.foreco.2004.11.017>.

Wessels, C.B., F.S. Malan, M. Kidd, and T. Rypstra. ‘The Variation of Microfibril Angle in South African Grown Pinus Patula and Its Influence on the Stiffness of Structural Lumber’. *Southern Forests* 77, no. 3 (2015): 213–19. <https://doi.org/10.2989/20702620.2015.1031575>.

Wheeler, J.A., S.D. Frey, and K.A. Stinson. ‘Tree Seedling Responses to Multiple Environmental Stresses: Interactive Effects of Soil Warming, Nitrogen Fertilization, and Plant Invasion’. *Forest Ecology and Management* 403 (2017): 44–51. <https://doi.org/10.1016/j.foreco.2017.08.010>.

Willis, J.L., and M.B. Walters. ‘Nutrition and Mycorrhizae Affect Interspecific Patterns of Seedling Growth on Coarse Wood and Mineral Soil Substrates’. *Ecosphere* 9, no. 7 (2018). <https://doi.org/10.1002/ecs2.2350>.

Willis, J.L., M.B. Walters, and K.W. Gottschalk. ‘Scarification and Gap Size Have Interacting Effects on Northern Temperate Seedling Establishment’. *Forest Ecology and Management* 347 (2015): 237–47. <https://doi.org/10.1016/j.foreco.2015.02.026>.

Wotton, D.M., and D. Kelly. ‘Frugivore Loss Limits Recruitment of Large-Seeded Trees’. *Proceedings of the Royal Society B: Biological Sciences* 278, no. 1723 (2011): 3345–54. <https://doi.org/10.1098/rspb.2011.0185>.

Wurst, S., N. Kaiser, S. Nitzsche, J. Haase, H. Auge, M.C. Rillig, and J.R. Powell. ‘Tree Diversity Modifies Distance-Dependent Effects on Seedling Emergence but Not Plant-Soil Feedbacks of Temperate Trees’. *Ecology* 96, no. 6 (2015): 1529–39. <https://doi.org/10.1890/14-1166.1>.

Wyman, T.E., S.A. Trewick, M. Morgan-Richards, and A.D.L. Noble. ‘Mutualism or Opportunism? Tree Fuchsia (Fuchsia Excorticata) and Tree Weta (Hemideina) Interactions’. *Austral Ecology* 36, no. 3 (2011): 261–68. <https://doi.org/10.1111/j.1442-9993.2010.02146.x>.

Xia, Q., M. Ando, and K. Seiwa. ‘Interaction of Seed Size with Light Quality and Temperature Regimes as Germination Cues in 10 Temperate Pioneer Tree Species’. *Functional Ecology* 30, no. 6 (2016): 866–74. <https://doi.org/10.1111/1365-2435.12584>.

Xu, H., J. Yu, L. You, S. Xiao, S. Nie, T. Li, G. Ye, and D. Lin. ‘Drought Resistance Evaluation of Casuarina Equisetifolia Half-Sib Families at the Seedling Stage and the Response of Five NAC Genes to Drought Stress’. *Forests* 13, no. 12 (2022). <https://doi.org/10.3390/f13122037>.

Yamagawa, H., S. Ito, and T. Nakao. ‘Restoration of Semi-Natural Forest after Clearcutting of Conifer Plantations in Japan’. *Landscape and Ecological Engineering* 6, no. 1 (2010): 109–17. <https://doi.org/10.1007/s11355-009-0088-1>.

Yamazaki, M., S. Iwamoto, and K. Seiwa. ‘Distance- and Density-Dependent Seedling Mortality Caused by Several Diseases in Eight Tree Species Co-Occurring in a Temperate Forest’. *Plant Ecology* 201, no. 1 (2009): 181–96. <https://doi.org/10.1007/s11258-008-9531-x>.

Yan, X.-F., S. Fang, C. Shi, Z.-H. Qiu, and Y.-F. Zhou. ‘Effects of Simulated Cotyledon Predation on the Seed Germination and Early Seedling Growth of Quercus Wutaishanica’. *Chinese Journal of Ecology* 33, no. 4 (2014): 973–81.

Yang, X., J. Bauhus, S. Both, T. Fang, W. Härdtle, W. Kröber, K. Ma, et al. ‘Establishment Success in a Forest Biodiversity and Ecosystem Functioning Experiment in Subtropical China (BEF-China)’. *European Journal of Forest Research* 132, no. 4 (2013): 593–606. <https://doi.org/10.1007/s10342-013-0696-z>.

Yang, X., S. Li, B. Shen, Y. Wu, S. Sun, R. Liu, R. Zha, and S.-L. Li. ‘Demographic Strategies of a Dominant Tree Species in Response to Logging in a Degraded Subtropical Forest in Southeast China’. *Annals of Forest Science* 75, no. 3 (2018). <https://doi.org/10.1007/s13595-018-0764-0>.

Yang, Y., M. Zhang, and X. Yi. ‘The Effects of Masting on Rodent-Mediated Seed Dispersal Interaction of Sympatric Tree Species’. *Forest Ecology and Management* 446 (2019): 126–34. <https://doi.org/10.1016/j.foreco.2019.05.035>.

Yang, Y.-L., S.A. Cushman, S.-C. Wang, F. Wang, Q. Li, H.-L. Liu, and Y. Li. ‘Genome-Wide Investigation of the WRKY Transcription Factor Gene Family in Weeping Forsythia: Expression Profile and Cold and Drought Stress Responses’. *Genetica* 151, no. 2 (2023): 153–65. <https://doi.org/10.1007/s10709-023-00184-y>.

Yao, J., Z. Song, C. Zhang, L. Meng, and X. Zhao. ‘Effects of Distance and Density Dependence on Seedling Growth in a Broadleaved Korean Pine Forest in Jiaohe of Jilin Province, Northeastern’. *Beijing Linye Daxue Xuebao/Journal of Beijing Forestry University* 41, no. 5 (2019): 108–17. <https://doi.org/10.13332/j.1000-1522.20190027>.

Yi, X., Y. Zhang, X. Wang, Y. Wang, and L. Ji. ‘Effects of Nitrogen on the Growth and Competition between Seedlings of Two Temperate Forest Tree Species’. *Scandinavian Journal of Forest Research* 30, no. 4 (2015): 276–82. <https://doi.org/10.1080/02827581.2014.1001781>.

Yin, J., F. Lin, E. De Lombaerde, Z. Mao, S. Liu, J. Ye, S. Fang, and X. Wang. ‘The Effects of Light, Conspecific Density and Soil Fungi on Seedling Growth of Temperate Tree Species’. *Forest Ecology and Management* 529 (2023). <https://doi.org/10.1016/j.foreco.2022.120683>.

Yu, H., X. Tang, N. Liu, H. Wang, Z. Zhang, C. Shao, G. Dong, and L. Qu. ‘Influences of Multiple Successive Heat Waves Combined with Water Control and Supplement on Photosynthetic Characteristics and Growth Rate of Phoebe Bournei Seedlings’. *Shengtai Xuebao* 43, no. 8 (2023): 3224–35. <https://doi.org/10.5846/STXB202201190183>.

Yuan, S., N. Liu, H. Ren, H. Zhang, and J. Wang. ‘Do Pioneer Species Enhance Early Performance of Native Species in Subtropical Shrublands? An Examination Involving Six Native Species in South China’. *Community Ecology* 20, no. 1 (2019): 53–63. <https://doi.org/10.1556/168.2019.20.1.6>.

Zeng, X., Y. Du, and Y. Vitasse. ‘Untangling Winter Chilling and Spring Forcing Effects on Spring Phenology of Subtropical Tree Seedlings’. *Agricultural and Forest Meteorology* 335 (2023). <https://doi.org/10.1016/j.agrformet.2023.109456>.

Zhang, H., Z. Wang, Q. Zeng, G. Chang, Z. Wang, and Z. Zhang. ‘Mutualistic and Predatory Interactions Are Driven by Rodent Body Size and Seed Traits in a Rodent-Seed System in Warm-Temperate Forest in Northern China’. *Wildlife Research* 42, no. 2 (2015): 149–57. <https://doi.org/10.1071/WR14211>.

Zhang, H., C. Yan, W.U. Shiqi, S.I. Junjie, Y.I. Xianfeng, L.I. Hongjun, and Z. Zhang. ‘Effects of Masting on Seedling Establishment of a Rodent-Dispersed Tree Species in a Warm-Temperate Region, Northern China’. *Integrative Zoology* 16, no. 1 (2021): 97–108. <https://doi.org/10.1111/1749-4877.12450>.

Zhang, M., J. Zhu, M. Li, G. Zhang, and Q. Yan. ‘Different Light Acclimation Strategies of Two Coexisting Tree Species Seedlings in a Temperate Secondary Forest along Five Natural Light Levels’. *Forest Ecology and Management* 306 (2013): 234–42. <https://doi.org/10.1016/j.foreco.2013.06.031>.

Zhang, M., J. Zhu, and Q. Yan. ‘Seed Germination of Pinus Koraiensis Siebold & Zucc. in Response to Light Regimes Caused by Shading and Seed Positions’. *Forest Systems* 21, no. 3 (2012): 426–38. <https://doi.org/10.5424/fs/2012213-02721>.

Zhang, Y.-F., C. Wang, S.-L. Tian, and J.-Q. Lu. ‘Dispersal and Hoarding of Sympatric Forest Seeds by Rodents in a Temperate Forest from Northern China’. *IForest* 7, no. 2 (2014): 70–74. <https://doi.org/10.3832/ifor1032-007>.

Zhang, Z., A. Mallik, J. Zhang, Y. Huang, and L. Zhou. ‘Effects of Arbuscular Mycorrhizal Fungi on Inoculated Seedling Growth and Rhizosphere Soil Aggregates’. *Soil and Tillage Research* 194 (2019). <https://doi.org/10.1016/j.still.2019.104340>.

Zhao, J., Y. Song, T. Sun, Z. Mao, C. Liu, L. Liu, R. Liu, L. Hou, and X. Li. ‘Response of Seed Germination and Seedling Growth of Pinus Koraiensis and Puercus Mongolica to Comprehensive Action of Warming and Precipitation’. *Shengtai Xuebao/ Acta Ecologica Sinica* 32, no. 24 (2012): 7791–7800. <https://doi.org/10.5846/stxb201204060479>.

Zheng, J., Q.-J. Ou, T.-J. Zhang, W.-J. Liang, B.-H. Li, and C.-L. Peng. ‘Can Allelopathy Be Used to Efficiently Resist the Invasion of Exotic Plants in Subtropical Forests?’ *BioInvasions Records* 8, no. 3 (2019): 487–99. <https://doi.org/10.3391/bir.2019.8.3.03>.

Zhu, J., L. Jiang, L. Chen, X. Jin, C. Xing, J. Liu, Y. Yang, and Z. He. ‘Tree Seedling Growth Allocation of Castanopsis Kawakamii Is Determined by Seed-Relative Positions’. *Frontiers in Plant Science* 14 (2023). <https://doi.org/10.3389/fpls.2023.1099139>.

Zywiec, M. ‘Seedling Survival under Conspecific and Heterospecific Trees: The Initial Stages of Regeneration of Sorbus Aucuparia, a Temperate Fleshy-Fruited Pioneer Tree’. *Annales Botanici Fennici* 50, no. 6 (2013): 361–71. <https://doi.org/10.5735/085.050.0611>.
